# Cannabidiol preserves astrocyte viability and attenuates injury-induced glial stress responses across zebra finch song control nuclei

**DOI:** 10.3389/fphar.2026.1972787

**Published:** 2026-10-09

**Authors:** Dylan A. Marshall, Karen A. Litwa, Ken Soderstrom

**Affiliations:** 1 Department of Pharmacology and Toxicology, Brody School of Medicine at East Carolina University, Greenville, NC, United States; 2 Department of Anatomy and Cell Biology, Brody School of Medicine and East Carolina Diabetes and Obesity Institute at East Carolina University, Greenville, NC, United States

**Keywords:** glial reactivity, lysosomal stress, neuroinflammation, neuroprotection, oxidative stress, songbird, TBI, astrocyte

## Abstract

Chronic neuroinflammation following traumatic brain injury (TBI) increases the risk of affective disorders and early-onset dementias. Existing anti-inflammatory therapies are limited by systemic side effects with chronic use and by poor brain penetration of newer biologics. Cannabidiol (CBD), a non-euphorigenic phytocannabinoid with a favorable safety profile and multi-target neuroprotective actions, is a promising alternative. Using a songbird microlesion model of learned vocal recovery after targeted damage to the premotor nucleus HVC, we previously showed that systemic CBD (10 mg/kg/day) reduces vocal disruption, inflammatory cytokines, oxidative stress, and synaptic loss. Here we tested the hypothesis that these benefits involve astrocytes. CBD (10 mg/kg/day IM for 7 days) preserved astrocyte viability, attenuated injury-induced lysosomal stress, suppressed reactivity markers (C3, S100A10, aromatase), and upregulated key metabolic and antioxidant transcripts (glutamine synthetase and GCLM) across the interconnected song-control nuclei HVC, RA, and Area X. These changes were accompanied by reduced neuronal apoptosis in lesioned HVC. The results identify astrocyte-associated responses as a candidate cellular substrate of CBD’s circuit-wide protective effects and support its translational potential to mitigate secondary injury and communication deficits after TBI.

## 1 Introduction

A cascade of secondary injury processes is initiated by traumatic brain injury (TBI) that can persist for months to years, leading to chronic neuroinflammation that drives synaptic loss, neuronal dysfunction, and increased risk of affective disorders and early-onset dementias (Brett et al., 2022; Komoltsev et al., 2022; Qu et al., 2026). Communication impairments after TBI include cognitive-communication disorder, and less often aphasia or dysarthria. These deficits are common after moderate–severe injury and are associated with reduced social participation and lower rates of return to work (O’Neil-Pirozzi et al., 2021; Togher et al., 2023). The present model is not intended to reproduce affective illness or dementia. It is used because focal injury to a well-mapped vocal sensorimotor circuit produces quantifiable communication deficits while engaging secondary inflammatory cascades that contribute to those longer-term risks. The underlying self-reinforcing inflammatory cascades are difficult to treat with conventional therapies. Standard anti-inflammatory drugs carry significant side effects with long-term use, while many biologic agents (e.g., monoclonal antibodies targeting cytokines) exhibit poor brain penetration, highlighting the urgent need for safe, CNS-accessible anti-inflammatory strategies suitable for chronic administration (Arbo et al., 2022).

Cannabidiol (CBD), a non-euphorigenic phytocannabinoid, is a promising potential treatment. It has established efficacy against childhood-onset epilepsies that are often accompanied by speech and language delays [1–5]. During clinical trials of CBD in childhood epilepsy disorders, caretaker reports of improved communication accompanied seizure control and motivated investigation of CBD’s effects on neural circuits supporting sensorimotor vocal learning (Alalawi et al., 2019; Hodges et al., 2022). Unlike Δ^9^-tetrahydrocannabinol (THC), CBD exerts broad neuroprotective and anti-inflammatory actions through multiple molecular targets [6–8], including modulation of glial responses via PPARγ, TRPV channels, and related pathways. Over now years of clinical experience, CBD has maintained an excellent safety record, and remains largely free of serious adverse effects (Thiele, 2022). These properties position CBD as a compelling candidate for addressing the problem of chronic neuroinflammation that is too often triggered by TBI.

Vocal communication is a sensorimotor skill shared by humans and few other species (Petkov and Jarvis, 2012). It is well modeled in songbirds such as the zebra finch. Song is acquired during a sensitive developmental period and continuously refined through sensorimotor feedback through adulthood (Doupe and Kuhl, 1999). The song control system comprises discrete, functionally interconnected nuclei, including the vocal premotor cortical-like nucleus HVC (used as a proper noun), the vocal motor nucleus Robust nucleus of the arcopallium (RA), and the striatal/basal ganglia homolog Area X of striatum (Area X, Figure 1, Reiner et al., 2004) that share key organizational and functional features with human speech and language circuits (Mooney, 2009; Petkov and Jarvis, 2012). Unilateral microlesions of HVC (∼10% ablation) produce transient disruptions of learned adult song that recover over ∼10 days via auditory-dependent relearning (Thompson and Johnson, 2007; Thompson et al., 2007; Alalawi et al., 2019; Hodges et al., 2022; Tripson et al., 2023). Prior dose-response studies from our laboratory demonstrated that CBD (10 mg/kg/day) robustly attenuates neuroinflammatory markers, oxidative stress, and synaptic loss in this model, with evidence of accelerated song recovery (Alalawi et al., 2019; Hodges et al., 2022; Tripson et al., 2023).

**FIGURE 1:**
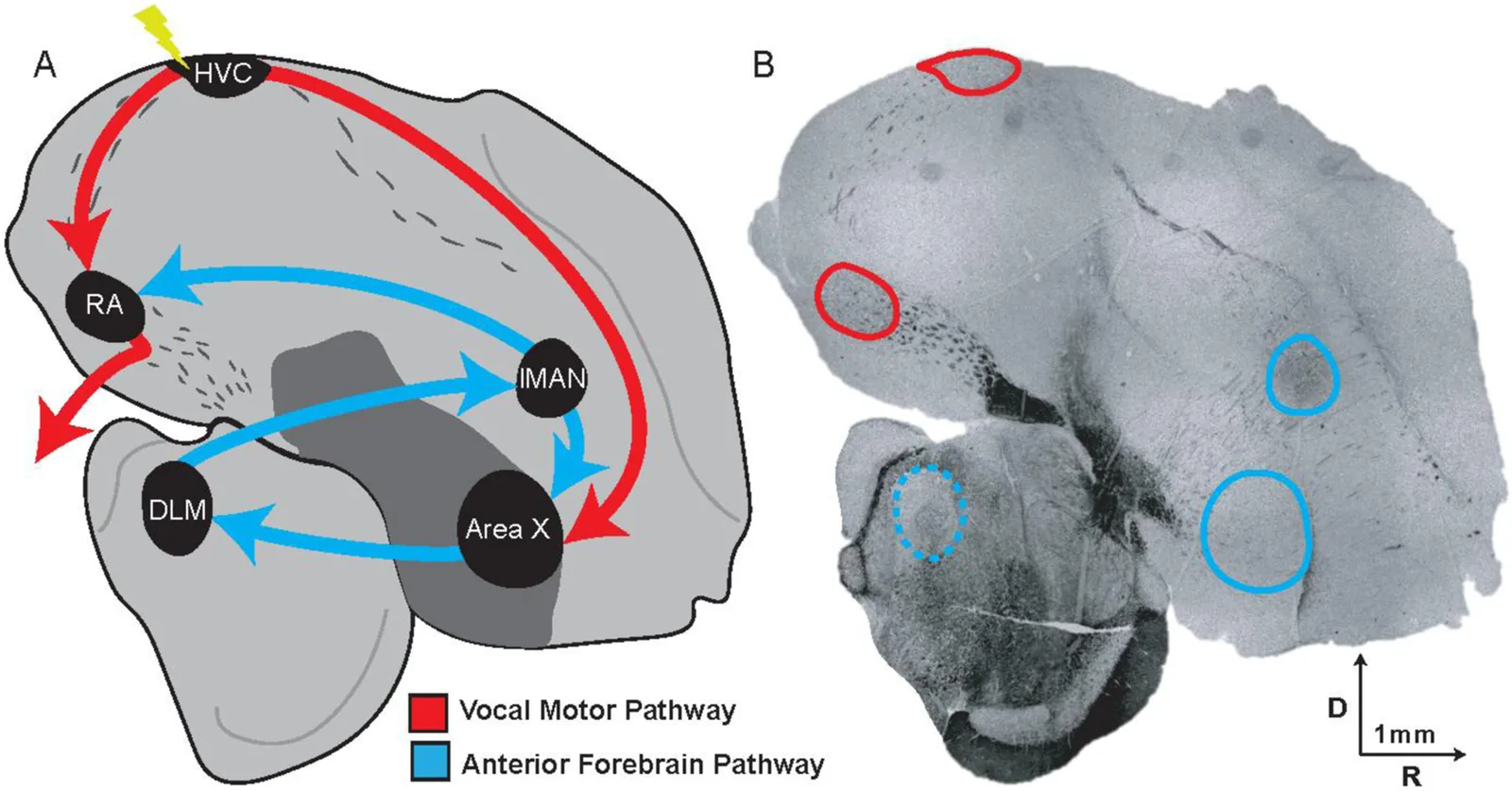
Schematic representation of the zebra finch song control system and experimental target region. **(A)** Illustration of song control nuclei studied and their major interconnections. Unilateral microlesions (yellow bolt) target the premotor cortical-like nucleus HVC, which projects to the vocal motor nucleus RA (robust nucleus of the arcopallium; red arrows, vocal motor pathway) and the striatal/basal ganglia nucleus Area X (blue arrows, anterior forebrain pathway [AFP], a cortico-basal ganglia-thalamic loop critical for vocal learning and sensorimotor maintenance). Additional AFP components include lMAN (lateral magnocellular nucleus of the anterior nidopallium) and DLM (medial portion of the dorsolateral thalamic nucleus; dashed outline indicates the region is not present in the section shown). **(B)** Representative coronal darkfield section used to identify song nuclei for lesioning, tissue microdissection, and immunohistochemical analyses. Rostral is to the right and dorsal is upward. Arrow = 1 mm. The distinct nuclear organization of song regions, compared with the laminated mammalian cortex, facilitates targeted lesioning, tissue collection, and histological analyses in this model.

Here we focus on responses within directly-lesioned HVC and two of its primary efferent targets, RA and Area X. This approach permits examination of both local lesion effects and potential post synaptic propagation of secondary injury processes across functionally integrated vocal learning circuitry. Building on prior findings, the present study examines the possible contribution of astrocytes to CBD-mediated neuroprotection. Astrocytes play important roles in regulating synaptic stability, redox balance, cytokine signaling, and neurotrophic support after CNS injury (Burda et al., 2016; Allen and Eroglu, 2017; Allen and Lyons, 2018; Verkhratsky and Nedergaard, 2018; Yuan and Wu, 2022; Eraso-Pichot et al., 2023). Because injury-induced astrocyte reactivity encompasses a range of functional states that can be either supportive or detrimental (Sofroniew and Vinters, 2010; Liddelow et al., 2017; Escartin et al., 2021), and because persistent neuroinflammation contributes to long-term disability after TBI, we hypothesized that CBD would reduce markers of astrocyte stress while supporting their homeostatic functions within the song circuit.

We found that CBD preserves astrocyte viability, attenuates injury-induced lysosomal stress markers, suppresses reactivity indicators, and increases expression of key metabolic and antioxidant enzymes (glutamine synthetase [GS] and glutamate-cysteine ligase modifier subunit [GCLM]) across HVC, RA, and Area X following focal injury. These astrocytic effects provide a glial link to the circuit-wide anti-inflammatory, antioxidant, and synaptoprotective actions of CBD observed previously and may support the accelerated recovery of complex learned vocal behavior reported in earlier studies. This recovery process shares fundamental mechanisms with the effects of TBI on human speech, language, and other sensorimotor skills.

## 2 Methods

### 2.1 Materials

Unless stated otherwise, materials and reagents were obtained from MilliporeSigma or Thermo-Fisher Scientific. Cannabidiol (CBD) was supplied by GW Research Ltd. (Cambridge, United Kingdom) as a crystalline powder with >98% purity. CBD was suspended in vehicle to achieve a final dosing of 10 mg/kg in 50 µL. The vehicle consisted of a 2:1:17 ratio of ethanol:Alkamuls EL-620 (Rhodia, Cranbury, NJ):phosphate-buffered saline. The resulting ethanol dosage was 0.33 mg/kg per day, which is lower than levels voluntarily self-administered by zebra finches (Olson et al., 2014). Isoflurane (Isospire, Dechra #17033-091–25), used for anesthesia, was provided by the Department of Comparative Medicine at East Carolina University. Bupivacaine (0.05%, ∼75 µL) was provided by the Department of Comparative Medicine at East Carolina University and used as a local anesthetic prior to surgery. Euthanasia was by isoflurane overdose followed by rapid decapitation or isoflurane anesthesia followed by overdose of pentobarbital sodium solution (Fatal-Plus, Vortech Pharmaceuticals, purchased from Patterson Veterinary) and rapid decapitation.

### 2.2 VEH and CBD treatments

VEH and CBD stock solutions, prepared as described above, were stored in sterile 5 mL septum-capped vials at 4 °C and refreshed at least once per week. For injections, drug solutions were loaded into sterile 1 cc insulin syringes fitted with 30-gauge needles. On injection days, birds were captured by hand in the early morning while lights remained off. The pectoralis muscle was exposed by parting the feathers with a small amount of 70% ethanol applied via squirt bottle. A 50 µL injection was administered into one of four quadrants of the pectoralis muscle, with the site rotated daily to reduce the risk of localized tissue damage from repeated dosing. Note that the 10 mg/kg/day dosage was selected based on a large, sham-controlled behavioral dose-response experiment that demonstrated it was the lowest fully-effective dosage employed, with efficacy equal to that of 100 mg/kg/day (Alalawi et al., 2019). We have since determined that CBD at 3 mg/kg/day is consistently less effective than 10 mg/kg/day in this model (Hodges et al., 2022).

### 2.3 Experimental timeline

Experiments were conducted over an 8-day period (see Figure 2 for a summary). Birds received six daily intramuscular injections of 50 μL CBD prior to surgery to allow the drug, given its lipophilic nature, large volume of distribution, and prolonged elimination half-life, to approach steady-state levels. On day 7, a final pre-operative injection was administered immediately before microlesion surgery so that tissue exposure spanned the perioperative period. The resulting 7-day regimen therefore comprised 6 days of pretreatment plus one pre-surgical dose. There were no post-injury treatments. Birds were euthanized 24 h later (day 8, note this single timepoint is a limitation of our study, and was not adequate for most birds to recover singing behavior). Brains were either perfused and fixed in paraformaldehyde or rapidly collected fresh for RNA isolation.

**FIGURE 2:**
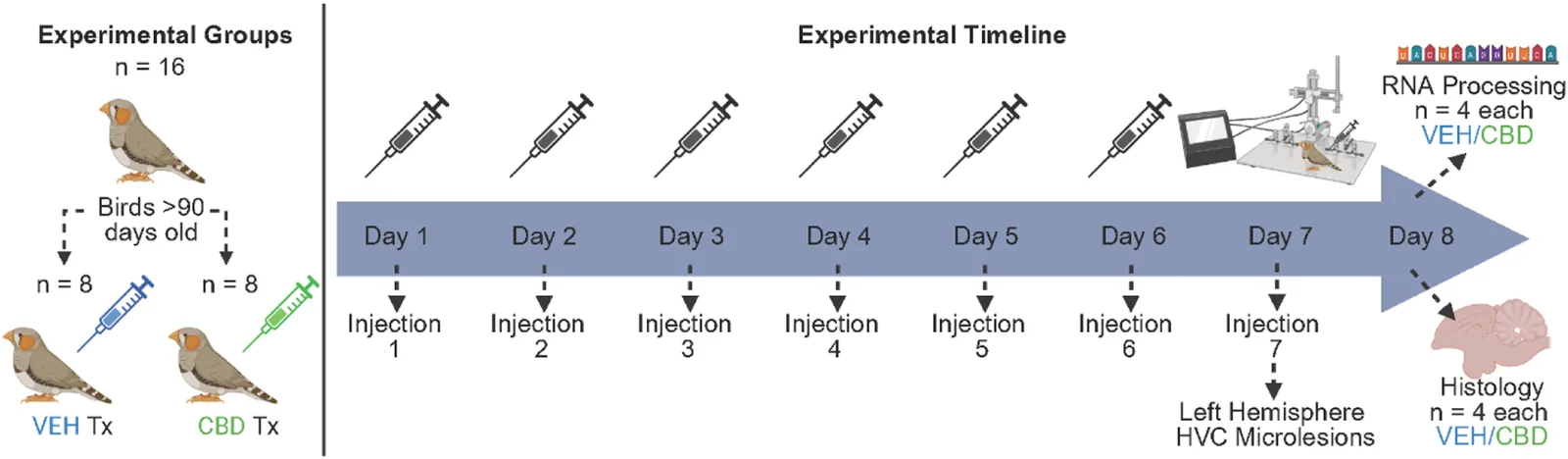
Experimental design and treatment timeline. Adult male zebra finches (>90 days post-hatch) were randomly assigned to vehicle (VEH) or cannabidiol (CBD) treatment groups. Animals received once-daily intramuscular injections (50 µL) for six consecutive days (Days 1–6), with CBD administered at 10 mg/kg. This pretreatment regimen was used to allow tissue CBD concentrations to approach steady-state levels before surgery. On Day 7, a final pre-operative injection was administered immediately before unilateral HVC microlesion surgery. Birds were euthanized 24 h post-surgery (Day 8) to capture acute injury responses. For histological analyses, birds were transcardially perfused with paraformaldehyde and brains were fixed for sectioning. For RNA analyses, brains were rapidly extracted and fresh tissue was microdissected from HVC, RA, and Area X for immediate RNA isolation.

### 2.4 Animals and recording environment

Adult male zebra finches (*Taeniopygia guttata*, ≥90 days post-hatch) were obtained from our breeding colony and housed under standard conditions (78°F, 12:12 h light/dark cycle) with *ad libitum* access to food and water. Only males were used because they produce learned song. Birds were individually housed in standard finch cages (9″× 11″× 17″). Visual isolation from conspecifics was maintained, although complete acoustic isolation was not enforced. Sound-attenuated chambers ensured unequivocal identification of recorded individuals. Vocal behavior was not the primary endpoint of this 24-h tissue study. To provide supplementary context for lesion effects on song, we analyzed historical recordings from prior projects that used the same vehicle or CBD regimen and the same 24-h post-lesion endpoint. Those archives included 30 recorded birds; 13 produced enough pre- and post-lesion song for analysis (VEH n = 7; CBD n = 6). This subset is not representative of the recorded cohort or of the immunofluorescence and qRT-PCR cohort, because most birds do not resume singing within 24 h. Only two of the 13 song-analysis birds, both vehicle-treated, were also used for PCR and immunohistochemistry. Song analyses used syllable segmentation in Sound Analysis Pro 2011 (Tchernichovski et al., 2000) together with a custom cluster-movement utility based on principal component analysis of syllable acoustics. Complete recording, segmentation, feature-extraction, clustering, and statistical procedures are provided in the Supplementary Methods.

Tissue for immunofluorescence (IF) and qRT-PCR analyses was collected from four adult male zebra finches per treatment group (vehicle or CBD). Because not all multiple markers, antibody panels, and endpoints could be assessed from individual animals, a larger total number of birds was required to obtain sufficient tissue for all planned assays. The original experimental design included two replicate cohorts of four birds each (total planned n = 8 per group). However, the use of unilateral lesions provided powerful within-subject contralateral controls, and molecular/cellular endpoints exhibit lower biological variability than behavioral measures. After the first cohort, effect sizes for the primary outcomes were sufficiently large (partial η_p^2^ = 0.37–0.98; see Supplementary Table S2) to provide high statistical power (>0.90 for detected effects). In accordance with the Reduction principle of the animal use 3Rs, the second replicate cohort was not performed. Statistical power considerations are presented in detail in Section 2.11.

### 2.5 HVC microlesion surgeries

Unilateral microlesions of the HVC were performed using previously established methods (Alalawi et al., 2019). Lesions were targeted to the left hemisphere based on evidence of lateralization patterns in zebra finches that resemble those seen in human language, highlighting the functional similarities between birdsong and speech (Moorman et al., 2012). Note that prior work demonstrated that left-hemisphere HVC lesions result in more pronounced vocal impairments compared to right-hemisphere lesions (Tripson et al., 2023). Prior to surgery, birds received bupivacaine (0.05%, ∼75 µL) as a local anesthetic and were anesthetized with isoflurane. Birds were positioned in a stereotaxic apparatus with the bifurcation of the midsagittal sinus serving as the stereotaxic zero. Small craniotomies were made above the left HVC. To achieve partial (∼10%) HVC damage, two lesion sites were targeted: 2.4 mm and 2.8 mm lateral to stereotaxic zero, each to a depth of 0.6 mm. Lesions were induced by applying 100 μA of current for 35 s at each site using a PFA-coated tungsten electrode (A-M Systems; 0.008 in bare diameter, 0.011 in coated diameter), with the insulation removed at the electrode tip to expose the tungsten at the lesion site. Birds recovered in a warmed incubator before being returned to their recording chambers.

### 2.6 Quantitative RT-PCR

As discussed above, cohorts of n = 4 birds were employed. cDNA samples were analyzed in triplicate (technical replicates). For each animal, 1 mm diameter biopsy punches were obtained from 2 mm thick parasagittal sections of the telencephalon (see Figure 1) using RNase-free techniques (sterile, RNase-free gloves and materials). Punches were used to isolate tissue from three brain regions of interest—HVC, RA, and Area X—from both hemispheres. These assays therefore measure bulk-region transcript abundance rather than astrocyte-specific expression; assignment of selected transcripts to astrocytes is inferential and is based on established cellular enrichment (GS, GCLM, C3, S100A10, aromatase) together with the cell-type-resolved immunofluorescence results. The unlesioned right hemisphere served as an internal control, while the lesioned left hemisphere represented the injury condition. Tissue samples were homogenized in TRIzol reagent (Invitrogen, 15-596–026), followed by phase separation with chloroform and RNA precipitation using isopropanol. The resulting RNA pellets were washed and resuspended in RNase-free water, and RNA concentration and purity were assessed using a NanoDrop spectrophotometer. Complementary DNA was synthesized from 250 ng of total RNA using an iScript cDNA synthesis kit (Bio-Rad, 1,708,890). Completed cDNA reactions were diluted five-fold using RNase-free water prior to quantitative PCR. Quantitative PCR was performed using SYBR Green Supermix (Bio-Rad, 1-725–271). Amplification specificity was verified by melt curve analysis, and cycle threshold (Ct) values were obtained using Design and Analysis Software version 2.8.0 (Thermo Fisher Scientific). Gene expression levels were normalized to the reference gene TBP, and relative expression was calculated using the ΔΔCt method with the unlesioned hemisphere as the comparator. Primer sequences are provided in Supplementary Table S1.

### 2.7 Tissue harvesting and processing

Following euthanasia and transcardial perfusion with phosphate-buffered saline followed by 4% paraformaldehyde (PFA), brains were post-fixed overnight in 4% PFA at 4 °C. Tissue was then cryoprotected in 20% sucrose until brains sank, indicating sufficient water displacement and infiltration. Brains were bisected at the midline, embedded in OCT compound, and rapidly frozen using a slurry of dry ice and 2-methylbutane. Parasagittal sections (10 µm) were cut using a cryostat maintained at −20 °C. Sections from both hemispheres were mounted onto Superfrost Plus slides and stored at −20 °C until further processing.

### 2.8 Immunofluorescent staining

Unless otherwise stated, all steps were performed at room temperature (25 °C). To reduce autofluorescence from lipofuscin, sections were treated with TrueBlack® Lipofuscin Autofluorescence Quencher (Cell Signaling Technology, Cat. #92401) according to the manufacturer’s instructions. Briefly, slides were incubated in TrueBlack working solution, rinsed thoroughly in phosphate-buffered saline (PBS), and allowed to equilibrate prior to downstream processing. Following autofluorescence quenching, sections were permeabilized in 0.2% Triton X-100 for 2 min and then blocked in 5% normal goat serum for 1 h. Primary antibodies were diluted in 2% normal goat serum and applied at optimized concentrations, including anti-glutamine synthetase (1:500; Synaptic Systems, Cat. #367004), anti-LAMP1 (1:500; Abcam, ab24170), anti-cleaved caspase-3 (Asp175,1:500; Cell Signaling Technology, Cat. #9661), and anti-NeuN (clone A60,1:500; Sigma-Aldrich, Cat. #ZMS377). Primary antibodies were applied in separate staining panels or in compatible combinations, depending on host species and fluorophore compatibility. Slides were incubated with primary antibodies overnight at 4 °C. The next day, slides were incubated with secondary antibodies—Alexa Fluor 647 goat anti-mouse IgG (H + L) (Thermo Fisher Scientific Cat. #A-21235 1:500) Alexa Fluor 555 goat anti-guinea pig IgG (H + L) (Thermo Fisher Scientific Cat. #A-21435 1:500) and Alexa Fluor 488 goat anti-rabbit IgG (H + L) (Thermo Fisher Scientific Cat. #A-11008 1:500)—diluted in 2% normal goat serum for 1 h. After secondary incubation, slides were washed twice with PBS (5 min each), counterstained with Hoechst (1:10,000) and washed a final time. Coverslips were mounted using Fluoro-Gel with Tris Buffer mounting medium (Electron Microscopy Sciences).

### 2.9 Fluorescent imaging

Regions of interest included HVC (excluding infarcted areas), RA, and Area X. To ensure consistent anatomical representation across treatment conditions, sections containing portions of all three regions were selected for imaging. Fluorescent images were acquired using a Keyence BZ-X800 fluorescence microscope equipped with a ×20 Plan Apo objective (NA 0.75; Keyence, model BZ-PA20). Images were captured using DAPI, GFP, TXRED, and Cy5 filter sets. For each region, three non-overlapping images were collected to ensure representative sampling across the area and to minimize localized variability. Image thresholding parameters were held constant across all images to ensure uniform quantification.

### 2.10 Image analysis

Image analysis was performed using ImageJ software (NIH). Fluorescent images were converted to 8-bit grayscale and thresholded using consistent parameters across all animals and conditions. For cell identification and cleaved caspase-3 (CC3) analysis, Hoechst-stained nuclei were thresholded using the default method, segmented using the watershed function, and subjected to particle analysis with a size constraint of 10–300 μm^2^ to identify individual nuclei. Nuclear masks were applied to additional channels, and logical AND operations were used to assess marker overlap. Nuclei overlapping NeuN or glutamine synthetase (GS) signal were classified as neuronal or astrocytic, respectively, and nuclei overlapping CC3 signal were classified as apoptotic. Cell counts were expressed as the percentage of total Hoechst-positive nuclei per 20× field (10–300 μm^2^ nuclear size constraint) for each cell type (neurons: NeuN^+^; astrocytes: GS^+^). CC3-positive cells were expressed as the percentage of the corresponding cell type (i.e., %CC3^+^ of NeuN^+^ neurons or %CC3^+^ of GS^+^ astrocytes). Lysosomal measurements were expressed as average lysosome size (µm^2^) within GS-defined astrocyte ROIs (0–30 μm^2^ particle size constraint). All values were obtained from three non-overlapping fields per region per hemisphere using consistent thresholding across animals and conditions.

### 2.11 Statistical analysis

Statistical analyses were performed using GraphPad Prism version 11.0.0. Immunofluorescence (IF) data were analyzed using three-way mixed-effects models using restricted maximum likelihood (REML) estimation. Models included Bird ID as a random factor, Region (HVC, RA, Area X) × Lesion Condition (lesioned hemisphere or un-lesioned internal control) × Treatment (Vehicle or CBD), with dependent variables within Bird ID and across region treated as repeated measures where appropriate. When at least one interaction involving Region was statistically significant, data were stratified by region and *post hoc* analyses were performed using two-way ANOVA (Lesion Condition × Treatment) within each region, with multiple comparisons corrected using Tukey’s test. Quantitative RT-PCR gene expression data were analyzed using two-way mixed-effects models (Region × Treatment), with region treated as a repeated-measures factor within subjects, followed by Sidak’s multiple comparisons correction. IF data are presented as estimated means ± standard deviation (SD), while qRT-PCR data are presented as estimated means ± standard error of the mean (SEM). A p-value ≤0.05 was considered statistically significant. Complete statistics, including fixed effects from mixed-effects models (Supplementary Table S2) and *post hoc* multiple comparisons (Supplementary Table S3), are provided in the Supplementary Material.

### 2.12 Effect size and statistical power

Effect sizes were calculated as partial eta squared (η_p^2^) using the formula η_p^2^ = (F × df_numerator)/(F × df_numerator +df_denominator). Across primary outcomes, partial η_p^2^ values ranged from moderate to very large (0.37–0.98 for key Treatment main effects and interactions; see Supplementary Table S2 for all values), demonstrating substantial biological relevance of the CBD and lesion effects. These large effect sizes, combined with the efficient within-subject contralateral control design and repeated-measures structure of the mixed-effects models, provided high statistical power (>0.90 for all detected effects) despite the modest sample size of n = 4 birds per group. This approach is consistent with our prior studies using this model and aligns with the principles of Reduction in the 3Rs. Accordingly, a second replicate cohort was not pursued, as it would have required doubling vertebrate animal impact with minimal additional scientific benefit.

### 2.13 Ethics approval statement

All animal procedures were approved by the East Carolina University Institutional Animal Care and Use Committee (IACUC protocol #W263) and the U.S. Army Animal Care and Use Review Office (ACURO). Experiments were conducted in accordance with institutional and national guidelines for the care and use of laboratory animals, and in compliance with the ARRIVE guidelines.

## 3 Results

### 3.1 CBD increases astrocyte percentages in a region-dependent manner

We first examined whether cannabidiol (CBD) alters astrocyte populations across song nuclei, given the central role of astrocytes in coordinating inflammatory and metabolic responses following injury. Representative immunofluorescence images from HVC, RA, and Area X are shown in Figures 3–5, and corresponding quantification is presented in Figure 6A. A three-way mixed-effects model revealed significant main effects of region (p < 0.0001) and treatment (p = 0.0004), and interactions including Region × Lesion condition (p = 0.0039) and Region × Treatment (p = 0.006) for astrocyte percentages (i.e., GS^+^/Hoechst^+^ nuclei expressed as a percentage of total Hoechst-labeled nuclei; see Supplementary Table S2 for complete three-way model statistics; Supplementary Table S3 for complete *post hoc* comparisons). Glutamine synthetase (GS) was selected as the primary astrocyte marker because it is widely and reliably expressed across pallial astrocytes in zebra finches, whereas glial fibrillary acidic protein (GFAP) is expressed only sparsely in the songbird pallium and is largely restricted to white matter and perivascular regions (Kálmán, 2025; Hinestroza-Morales et al., 2026). This makes GS a more appropriate and comprehensive marker for quantifying astrocyte populations and their responses in the zebra finch song control circuit.

**FIGURE 3:**
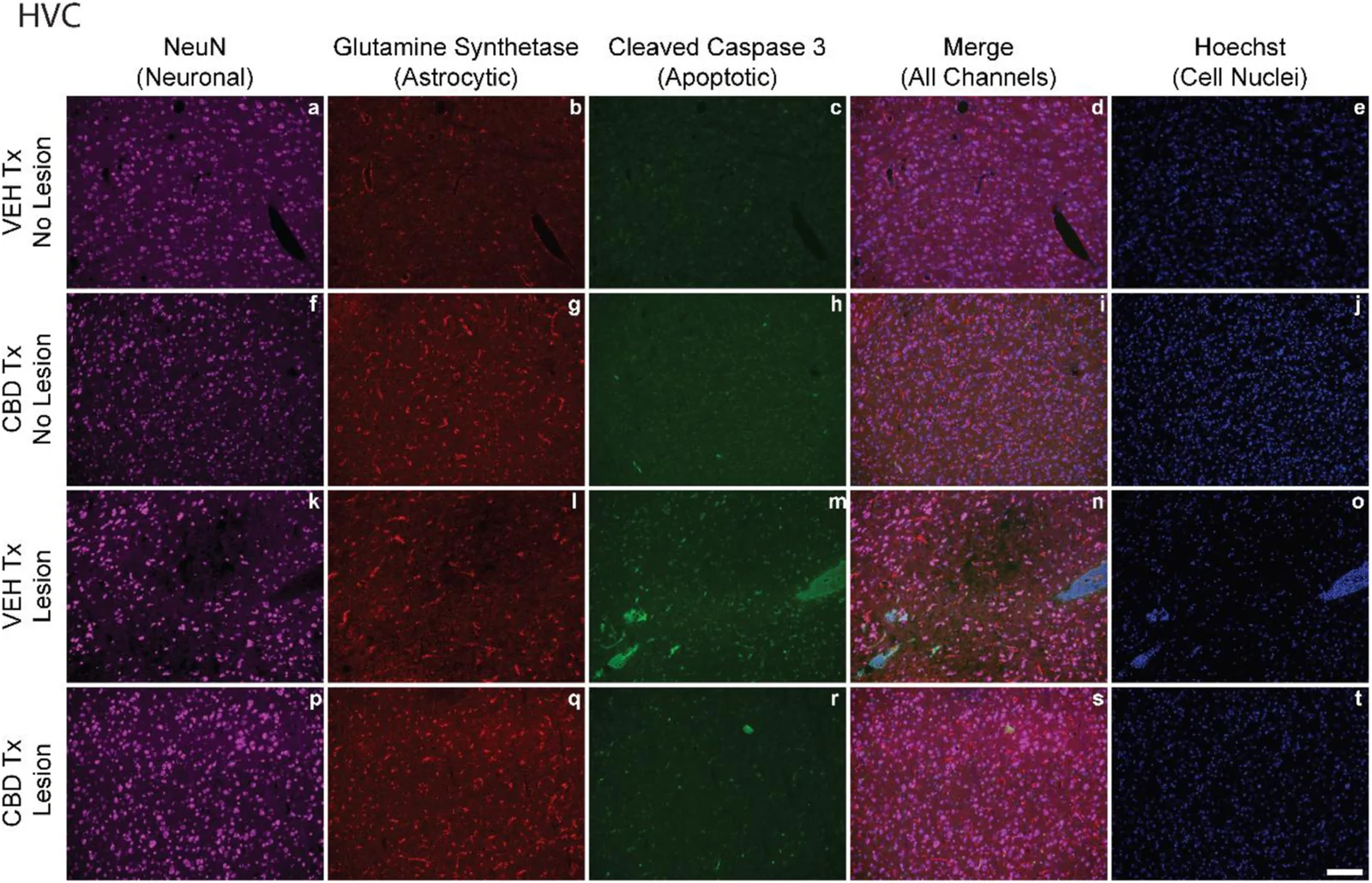
Cannabidiol preserves astrocyte viability and reduces injury-induced apoptosis in HVC following unilateral microlesions. Representative immunofluorescence images of NeuN (neuronal marker, magenta), glutamine synthetase (GS; astrocytic marker, red), cleaved caspase-3 (CC3; apoptotic marker, green), merged channels, and Hoechst nuclear counterstain (blue) in the lesioned and unlesioned hemispheres of VEH- and CBD-treated zebra finches. Scale bar = 200 µm. Quantitative analysis (see Figure 6 for statistical analyses across regions) depicts cell populations expressed as percentages of Hoechst^+^ nuclei. Representative high-magnification images demonstrating CC3 localization within GS-positive astrocytes are shown in Supplementary Figure S4. CBD treatment significantly preserved GS^+^ astrocytes and reduced GS^+^/CC3^+^ apoptotic astrocytes in HVC, with modest preservation of NeuN^+^ neurons.

**FIGURE 4:**
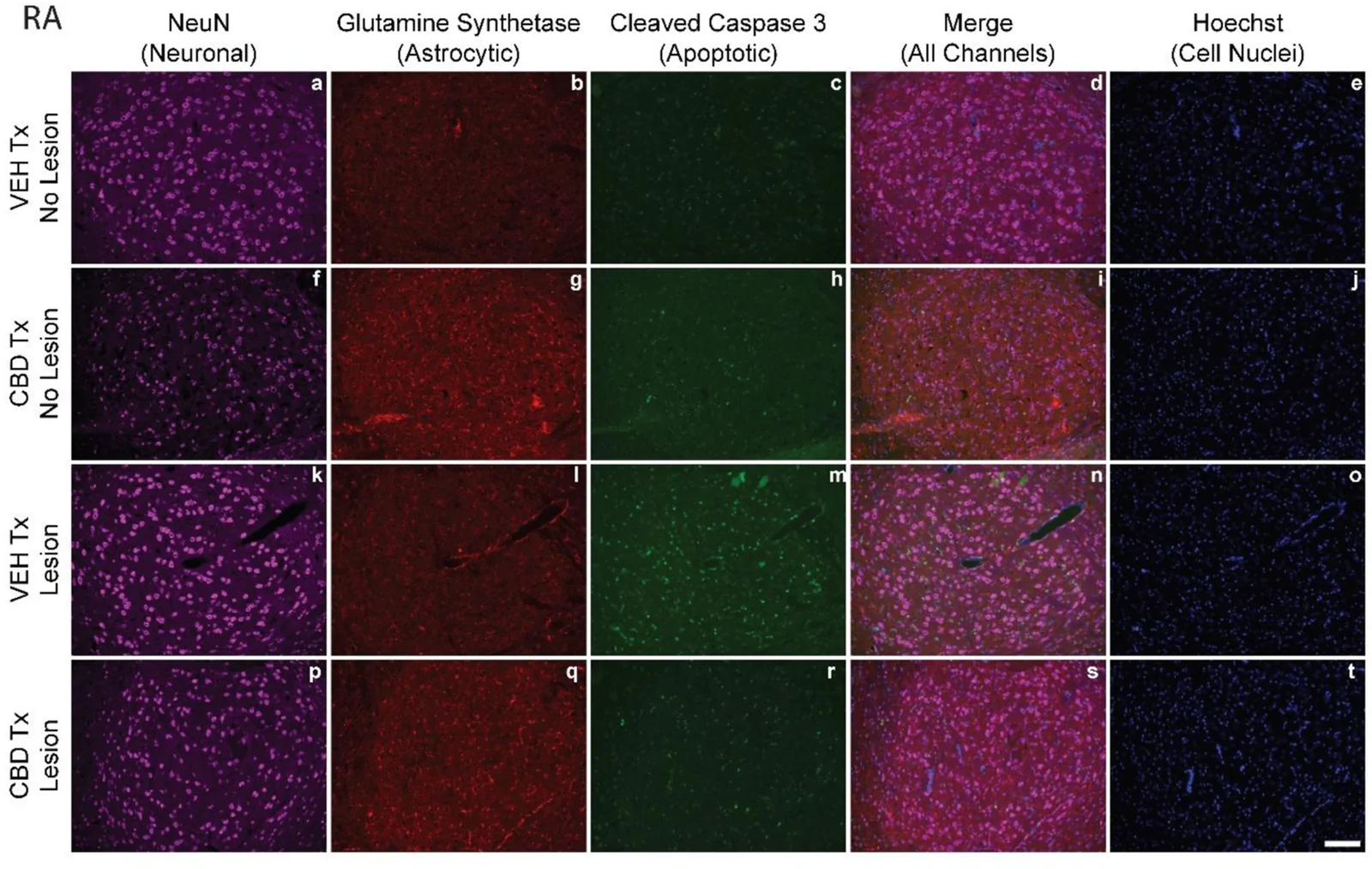
Cannabidiol preserves astrocyte viability and reduces injury-induced apoptosis in RA following unilateral HVC microlesions. Representative immunofluorescence images of NeuN (neuronal marker, magenta), glutamine synthetase (GS; astrocytic marker, red), cleaved caspase-3 (CC3; apoptotic marker, green), merged channels, and Hoechst nuclear counterstain (blue) in the lesioned and unlesioned hemispheres of VEH- and CBD-treated zebra finches. Scale bar = 200 µm. Quantitative analysis (see Figure 6 for statistical analyses across regions) depicts cell populations expressed as percentages of Hoechst^+^ nuclei. Representative high-magnification images demonstrating CC3 localization within GS-positive astrocytes are shown in Supplementary Figure S4. CBD treatment significantly preserved GS^+^ astrocytes and reduced GS^+^/CC3^+^ apoptotic astrocytes in RA.

**FIGURE 5:**
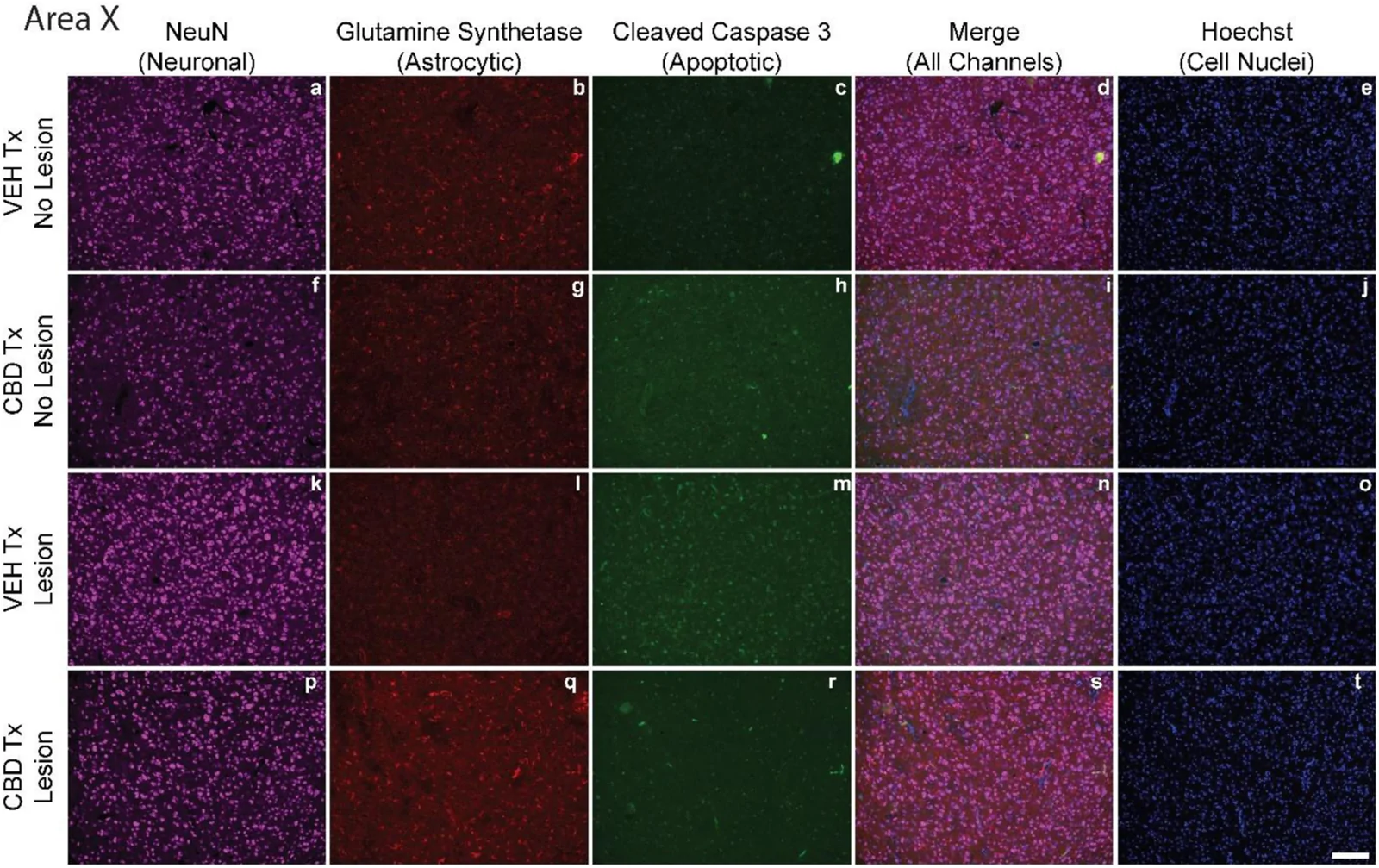
Cannabidiol preserves astrocyte viability and reduces injury-induced apoptosis in Area X following unilateral HVC microlesions. Representative immunofluorescence images of NeuN (neuronal marker, magenta), glutamine synthetase (GS; astrocytic marker, red), cleaved caspase-3 (CC3; apoptotic marker, green), merged channels, and Hoechst nuclear counterstain (blue) in the lesioned and unlesioned hemispheres of VEH- and CBD-treated zebra finches. Scale bar = 200 µm. Quantitative analysis (see Figure 6 for statistical analyses across regions) depicts cell populations expressed as percentages of Hoechst^+^ nuclei. Representative high-magnification images demonstrating CC3 localization within GS-positive astrocytes are shown in Supplementary Figure S4. CBD treatment significantly preserved GS^+^ astrocytes in Area X.

**FIGURE 6:**
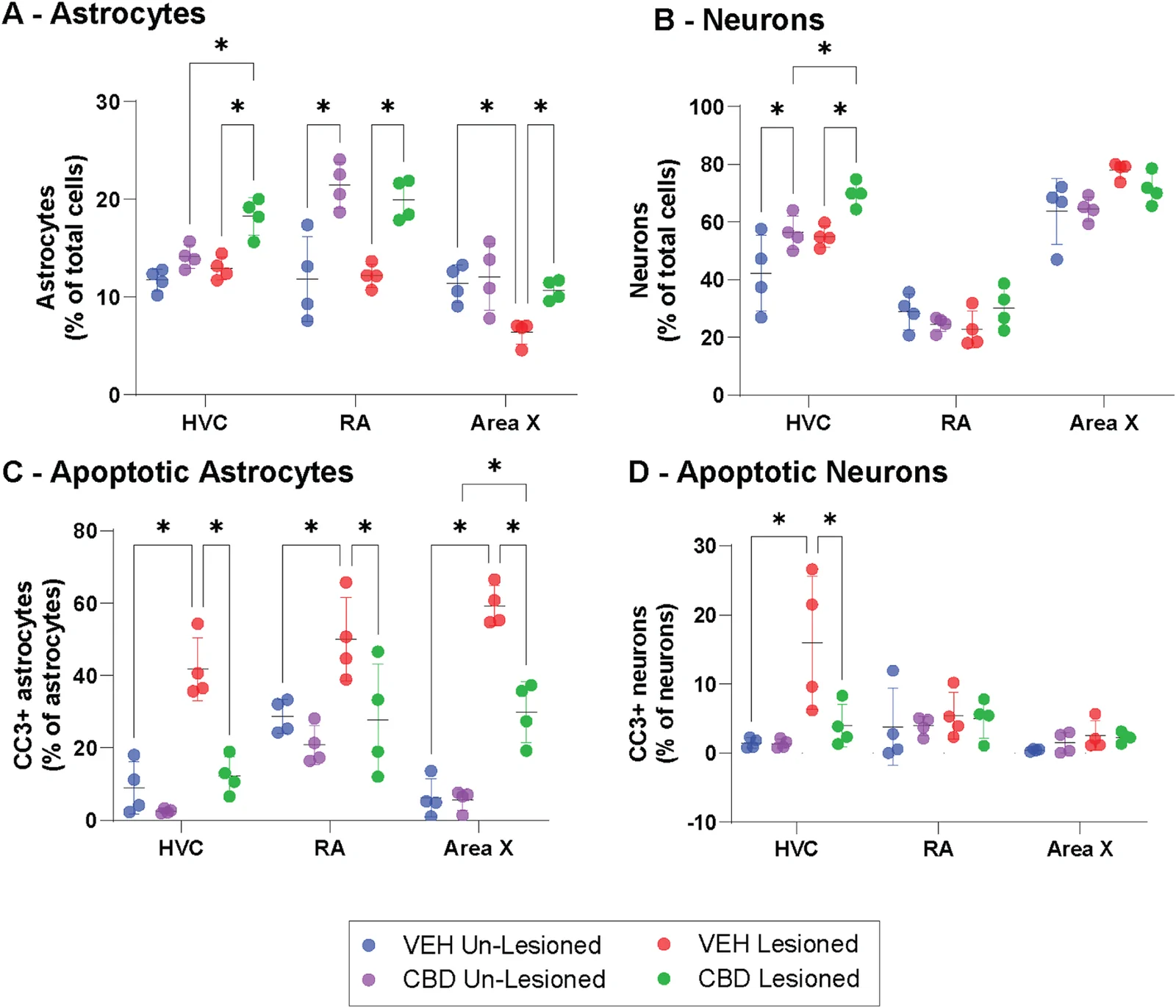
Quantitative analysis of astrocyte preservation, neuronal density, and apoptosis across song control nuclei following HVC microlesions. Summary of cell counts from the representative immunofluorescence images shown in Figures 3–5, expressed as percentages of Hoechst^+^ nuclei in HVC, RA, and Area X from VEH- and CBD-treated zebra finches. **(A)** GS^+^ astrocytes as a percentage of total Hoechst^+^ cells. **(B)** NeuN^+^ neurons as a percentage of Hoechst^+^ cells. **(C)** GS^+^/CC3^+^ apoptotic astrocytes. **(D)** NeuN^+^/CC3^+^ apoptotic neurons. Data are presented as mean ± SD with individual data points representing biological replicates (n = 4 per group). CBD treatment significantly preserved GS^+^ astrocyte populations and reduced astrocyte apoptosis across regions in a region-dependent manner, with modest preservation of neuronal density primarily in HVC. Apoptotic neurons remained low and were not significantly altered outside the lesion target (HVC). Statistical comparisons were performed using three-way mixed-effects ANOVA followed by Tukey’s multiple comparisons test (see Supplementary Table S2 for full model statistics; Supplementary Table S3 for *post hoc* comparisons; *p* < 0.05).

The Region × Lesion interaction indicates that injury effects vary across song nuclei, consistent with region-specific susceptibility to secondary injury processes. The Region × Treatment interaction indicates that CBD effects are not uniform across song nuclei, suggesting region-dependent modulation of astrocyte responses. The Lesion × Treatment interaction indicates that CBD effects depend on injury status, supporting an injury-dependent mechanism by which CBD preferentially modulates astrocyte responses triggered by lesioning of tissue.

Consistent with these interactions, *post hoc* comparisons revealed that CBD increased the percentage of GS^+^/Hoechst^+^ nuclei on the lesioned side in HVC (VEH: 12.92% vs. CBD: 18.25%, 95% CI [−7.85%, −2.81%], p = 0.0003), across both hemispheres in RA (lesioned: VEH: 12.16% vs. CBD: 19.96%, 95% CI [−8.17%, −0.51%], p = 0.0036; unlesioned: VEH: 11.83% vs. CBD: 21.45%, 95% CI [−14.60%, −4.64%], p = 0.0007), and on the lesioned side in Area X (VEH: 6.34% vs. CBD: 10.68%, 95% CI [−12.78%, −2.81%], p = 0.0269), with no change on the unlesioned side in Area X (p > 0.05). Together, these findings indicate that CBD drives region- and injury-dependent increases in astrocyte representation, consistent with a role for astrocytes as a candidate cellular substrate of CBD-modulated responses to neural injury.

### 3.2 CBD attenuates lesion-induced astrocyte apoptosis across song nuclei

To determine whether these shifts reflect altered survival, we next assessed astrocyte apoptosis, defined as the percentage of GS^+^/Hoechst^+^ astrocytes positive for cleaved caspase-3 (CC3; see Figure 6C). A three-way mixed-effects model revealed significant main effects of region (p = 0.0005), lesion condition (p < 0.0001), and treatment (p = 0.0008), together with Region × Lesion condition (p = 0.0007) and Lesion condition × Treatment (p = 0.0027) interactions for astrocyte apoptosis (three-way model: Supplementary Table S2; *post hoc* comparisons; Supplementary Table S3). The Region × Lesion interaction indicates that injury-induced astrocyte apoptosis varies across song nuclei. The Lesion × Treatment interaction indicates that CBD effects depend on injury status, consistent with selective modulation of astrocyte survival in lesioned tissue.

Consistent with these interactions, *post hoc* comparisons revealed that lesions increased the percentage of apoptotic astrocytes in HVC (VEH: 8.95% in unlesioned hemispheres vs. 41.76% in lesioned hemispheres, 95% CI [−43.26%, −22.37%], p = 0.0002), RA (VEH: 28.75% vs. 50.00%, 95% CI [−42.57%, −0.065%], p = 0.0498), and Area X (VEH: 6.20% vs. 59.31%, 95% CI [−63.75%, −42.47%], p < 0.0001). CBD reduced the percentage of apoptotic astrocytes on the lesioned side in HVC (VEH: 41.76% vs. CBD: 12.32%, 95% CI [18.27%, 40.63%], p < 0.0001), RA (VEH: 50.00% vs. CBD: 27.72%, 95% CI [3.80%, 40.76%], p = 0.019), and Area X (VEH: 59.31% vs. CBD: 29.92%, 95% CI [18.83%, 39.96%], p < 0.0001). Together, these findings indicate that lesions robustly increase astrocyte apoptosis across song nuclei, whereas CBD attenuates this response in an injury-dependent manner.

### 3.3 CBD selectively increases percentages of neurons within HVC

Given the role of astrocytes in supporting neuronal maintenance within song nuclei (Figure 6B), we next assessed whether astrocyte-associated changes were accompanied by altered neuronal percentages, defined as NeuN^+^/Hoechst^+^ nuclei expressed as a percentage of total Hoechst^+^ nuclei. A three-way mixed-effects model revealed significant main effects of region (p < 0.0001) and lesion condition (p = 0.0075), along with Region × Lesion condition (p = 0.0116) and Region × Treatment (p = 0.0022) interactions for neuronal percentages (three-way model: Supplementary Table S2; *post hoc* comparisons; Supplementary Table S3). The Region × Lesion interaction indicates that injury-related changes in neuronal percentages vary across song nuclei. The Region × Treatment interaction indicates that CBD effects are region-specific, suggesting selective modulation of neuronal populations. Consistent with these interactions, *post hoc* comparisons revealed that CBD increased neuronal percentages in HVC on both the unlesioned side (VEH: 42.25% vs. CBD: 56.27%, 95% CI [−27.98%, −0.07%], p = 0.0489) and lesioned side (VEH: 54.93% vs. CBD: 69.72%, 95% CI [−28.74%, −0.83%], p = 0.0378), with no effect detected in RA or Area X (p > 0.05). These findings indicate that CBD selectively increases neuronal percentages in HVC in a region-specific manner.

### 3.4 CBD reduces lesion-associated neuronal apoptosis in HVC

We then examined neuronal apoptosis, expressed as the percentage of neurons positive for cleaved caspase-3 (CC3^+^/NeuN^+^/Hoechst^+^ nuclei expressed as a percentage of total NeuN^+^/Hoechst^+^ nuclei), to determine whether injury and treatment effects on neurons paralleled those observed in astrocytes (Figure 6D). A three-way mixed-effects model revealed significant main effects of region (p = 0.0177) and lesion condition (p = 0.0267), together with Region × Lesion condition (p = 0.0039), Region × Treatment (p = 0.0379), and Region × Lesion condition × Treatment (p = 0.0212) interactions for neuronal apoptosis (three-way model: Supplementary Table S2; *post hoc* comparisons; Supplementary Table S3). The Region × Lesion interaction indicates that injury-induced neuronal apoptosis varies across song nuclei. The Region × Treatment and three-way interactions indicate that CBD effects on neuronal apoptosis are region- and injury-dependent. Consistent with these interactions, *post hoc* comparisons revealed that lesion increased the percentage of apoptotic neurons in HVC (VEH: 1.47% vs. 15.99%, 95% CI [−25.04%, −3.984%], p = 0.013), and that CBD reduced the percentage of apoptotic neurons on the lesioned side in HVC (VEH: 15.99% vs. CBD: 3.96%, 95% CI [2.836%, 21.22%], p = 0.0117), with no effects detected in RA or Area X (p > 0.05). These findings indicate that lesion-induced neuronal apoptosis was highest in HVC and was selectively attenuated by CBD in this region.

### 3.5 CBD attenuates lysosomal area following injury

We next examined lysosomal structure, as indicated by LAMP1^+^ puncta area within GS-defined astrocyte ROIs as an indicator of cellular stress (Figure 7). Lysosomal-associated membrane protein 1 (LAMP1) was used as a marker of lysosomal membranes because it is a highly validated, widely expressed component of the lysosomal limiting membrane and is commonly employed to assess lysosomal abundance, size, and stress responses in astrocytes and other glial cells following CNS injury (Settembre et al., 2013).

**FIGURE 7:**
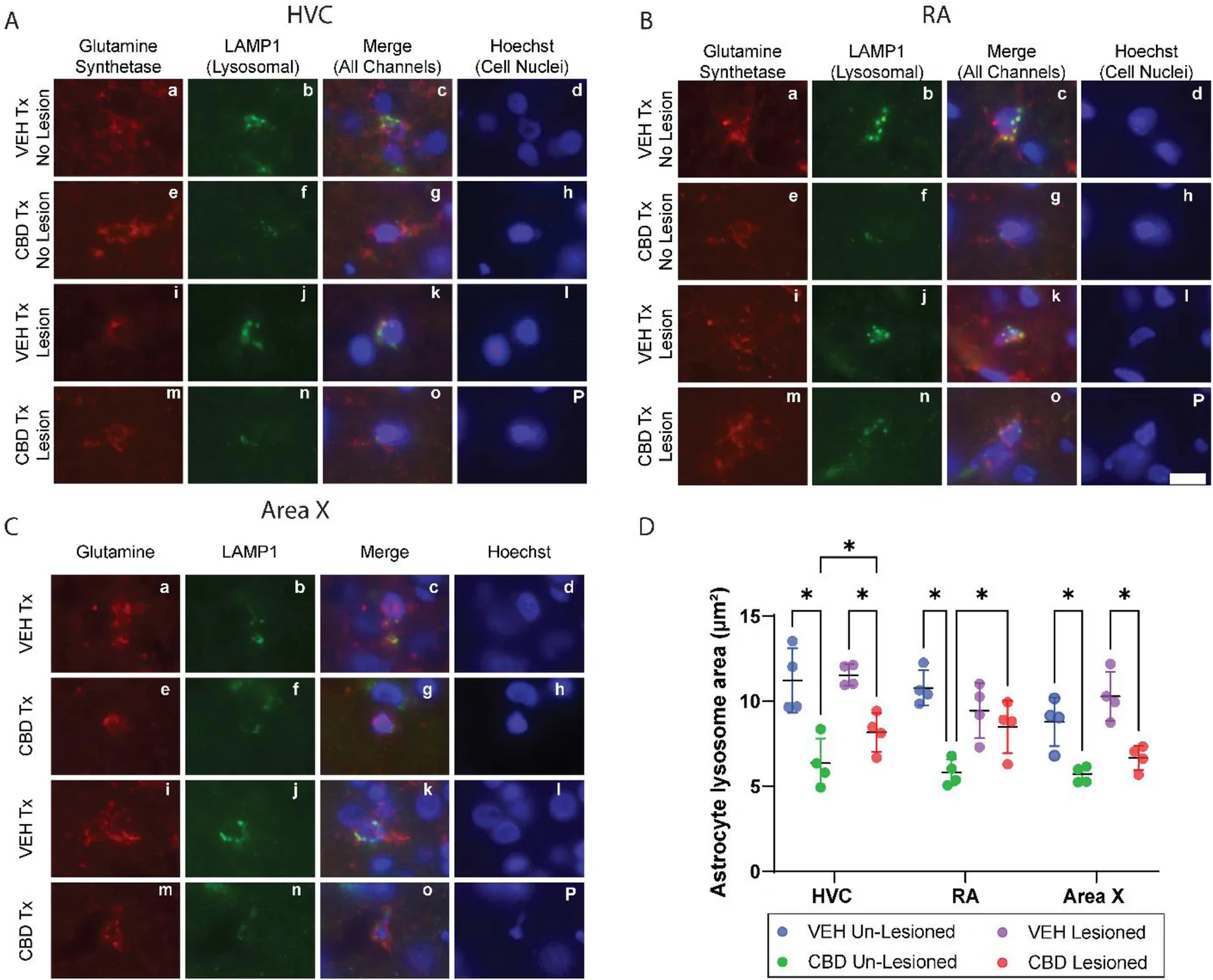
Cannabidiol reduces astrocytic lysosomal size in song control nuclei following injury. Representative immunofluorescence images from HVC **(A)**, RA **(B)**, and Area X **(C)** in the unlesioned and lesioned hemispheres of VEH- and CBD-treated birds. Astrocytes were labeled with glutamine synthetase (GS; red), lysosomes with LAMP1 (green), and nuclei with Hoechst (blue); merged images are shown. Representative high-magnification images illustrating LAMP1 localization within GS-positive astrocytes are shown in Supplementary Figure S3. Scale bar = 10 µm. **(D)** Quantitative analysis of mean LAMP1^+^ puncta area within GS^+^ astrocytes (µm^2^). Data are presented as mean ± SD with individual data points representing biological replicates (n = 4 per group). CBD treatment significantly reduced lysosomal size bilaterally across HVC, RA, and Area X compared with VEH-treated birds. In the lesioned RA, CBD-treated animals showed reduced lysosomal size relative to VEH-treated animals, although this difference did not reach statistical significance. Statistical comparisons were performed using mixed-effects ANOVA followed by Tukey’s multiple comparisons test (*p* < 0.05).

A three-way mixed-effects model revealed a significant Region × Lesion condition × Treatment interaction (p = 0.017; Supplementary Table S2). This interaction indicates that CBD effects on lysosome size are both region- and injury-dependent. Consistent with this, *post hoc* comparisons revealed that CBD reduced lysosome area in HVC within both the unlesioned side (VEH: 11.22 µm^2^ vs. CBD: 6.36 µm^2^, 95% CI [2.41 µm^2^, 7.31 µm^2^], p = 0.0006) and lesioned side (VEH: 11.53 µm^2^ vs. CBD: 8.17 µm^2^, 95% CI [0.91 µm^2^, 5.81 µm^2^], p = 0.0088), in RA on the unlesioned side (VEH: 9.44 µm^2^ vs. CBD: 8.49 µm^2^, 95% CI [2.649 µm^2^, 7.297 µm^2^], p = 0.0003) but not the lesioned side (p > 0.05), and in Area X on both the unlesioned side (VEH: 8.78 µm^2^ vs. CBD: 5.73 µm^2^, 95% CI [1.07 µm^2^, 5.03 µm^2^], p = 0.0039) and lesioned side (VEH: 10.28 µm^2^ vs. CBD: 6.67 µm^2^, 95% CI [1.64 µm^2^, 5.59 µm^2^], p = 0.0011). Together, these findings indicate that CBD broadly attenuates lysosomal area across song nuclei, with a reduced effect in the lesioned RA.

### 3.6 CBD reduces lysosomal and autophagy-associated gene expression

To determine whether the observed changes in lysosomal area were accompanied by transcriptional responses, we next quantified mRNA expression in bulk HVC, RA, and Area X punches using qRT-PCR, expressed as fold change relative to each animal’s contralateral control hemisphere (Figure 8). A two-way mixed-effects model revealed significant main effects of region and treatment, as well as a Region × Treatment interaction for LAMP1 mRNA expression (two-way model: Supplementary Table S2; *post hoc* comparisons; Supplementary Table S3). The Region × Treatment interaction indicates that CBD effects on lysosomal gene expression are region-dependent. Consistent with this, *post hoc* comparisons revealed that CBD reduced LAMP1 mRNA expression in HVC (VEH: 2.42-fold vs. CBD: 1.70-fold, 95% CI [0.415-fold, 1.032-fold], p < 0.0001) and RA (VEH: 1.14-fold vs. CBD: 0.53-fold, 95% CI [0.30-fold, 0.91-fold], p = 0.0002), with no effect in Area X (p > 0.05).

**FIGURE 8:**
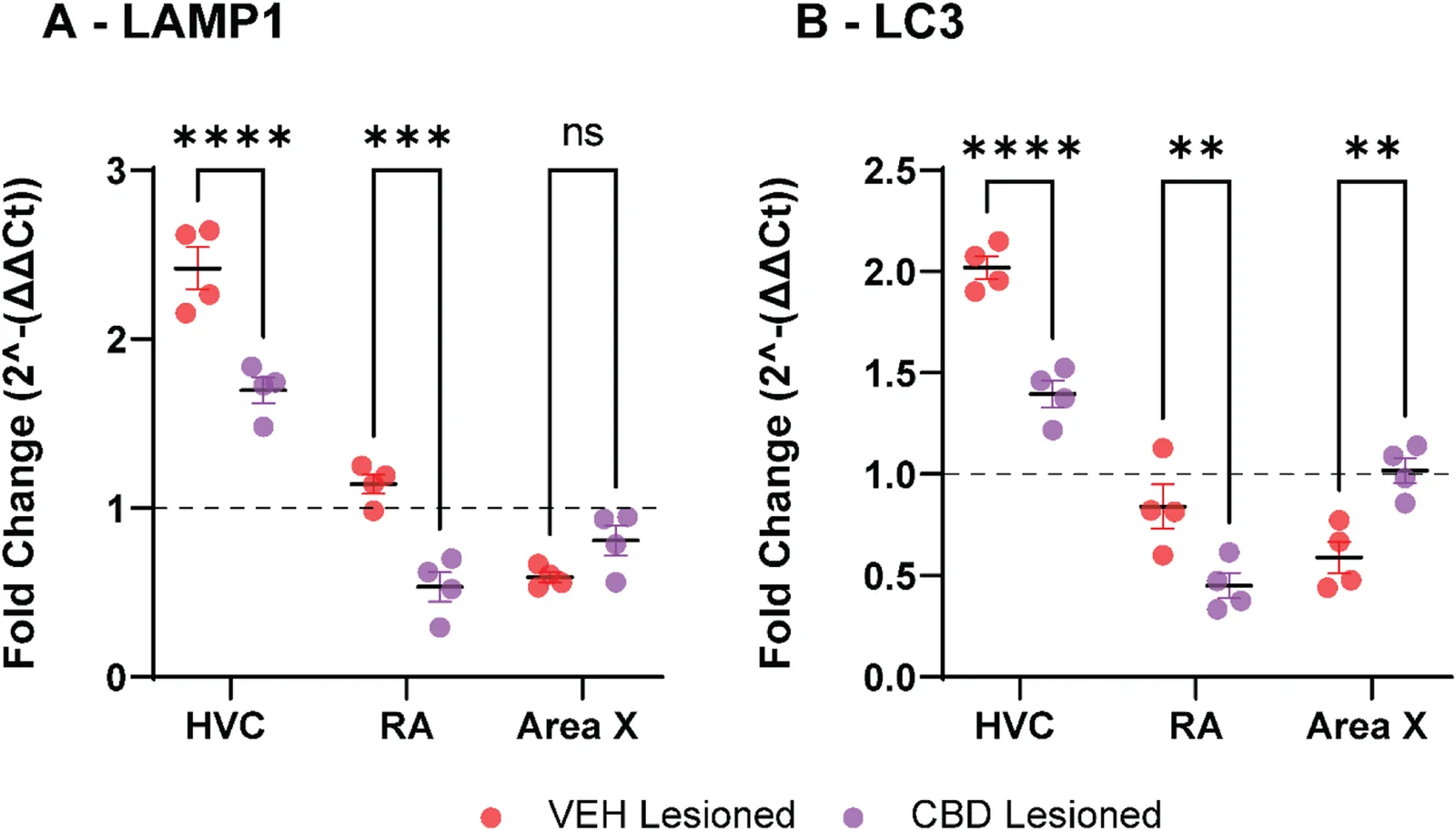
Cannabidiol modulates expression of lysosomal and autophagosomal markers in song control nuclei following injury. Quantitative RT-PCR analysis of lysosomal- and autophagosomal-related transcripts in the lesioned hemispheres of HVC, RA, and Area X from VEH- and CBD-treated birds, normalized to the contralateral unlesioned hemisphere. **(A)** LAMP1 (lysosomal membrane marker) mRNA expression was significantly reduced in HVC and RA following CBD treatment, with no significant change in Area X. **(B)** LC3 (autophagosomal marker) mRNA expression was significantly reduced in HVC and RA but significantly increased in Area X following CBD treatment. Data are presented as mean fold change ±SEM with individual data points representing biological replicates (n = 4 per group). Statistical comparisons were performed using mixed-effects ANOVA followed by Sidak’s multiple comparisons test (*p* < 0.05).

We next quantified mRNA expression of LC3, a marker of autophagosome formation, because it is the most widely validated indicator of autophagic activity (Klionsky et al., 2021). During autophagy, cytosolic LC3-I is lipidated to form LC3-II, which incorporates into autophagosomal membranes. Using a model for LC3 similar to that of LAMP1 described above, mRNA expression revealed significant effects of region (p < 0.0001), treatment (p = 0.0047), and their interaction (p < 0.0001; Figure 8B). The Region × Treatment interaction indicates that CBD effects on autophagy-related gene expression are also region-dependent. Post hoc comparisons revealed that CBD reduced LC3 mRNA expression in HVC (VEH: 2.020-fold vs. CBD: 1.394-fold, 95% CI [0.35-fold, 0.90-fold], p < 0.0001) and RA (VEH: 0.84-fold vs. CBD: 0.45-fold, 95% CI [0.11-fold, 0.67-fold], p = 0.0048), while increasing expression in Area X (VEH: 0.59-fold vs. CBD: 1.02-fold, 95% CI [−0.71-fold, −0.15-fold], p = 0.0022). Together, these findings indicate that CBD modulates lysosomal and autophagy-related transcriptional responses in a region-dependent manner.

### 3.7 CBD enhances astrocyte metabolic and antioxidant gene expression

We next examined levels of astrocyte-associated metabolic and antioxidant transcripts, given the central role of astrocytes in glutamate clearance and redox regulation following injury (Figure 9). Glutamine synthetase (GS) mRNA was quantified as a key marker of astrocytic metabolic function because it encodes the primary enzyme responsible for glutamate recycling into glutamine, thereby limiting excitotoxicity and supporting synaptic homeostasis and neuronal metabolic coupling (Allen and Eroglu, 2017; Allen and Lyons, 2018; Verkhratsky and Nedergaard, 2018). Glutamate-cysteine ligase modifier subunit (GCLM) mRNA was assessed as a marker of antioxidant capacity because it regulates the rate-limiting step in glutathione synthesis, a major cellular antioxidant that protects against oxidative stress in the injured CNS (Atalay Ekiner et al., 2022; Eraso-Pichot et al., 2023).

**FIGURE 9:**
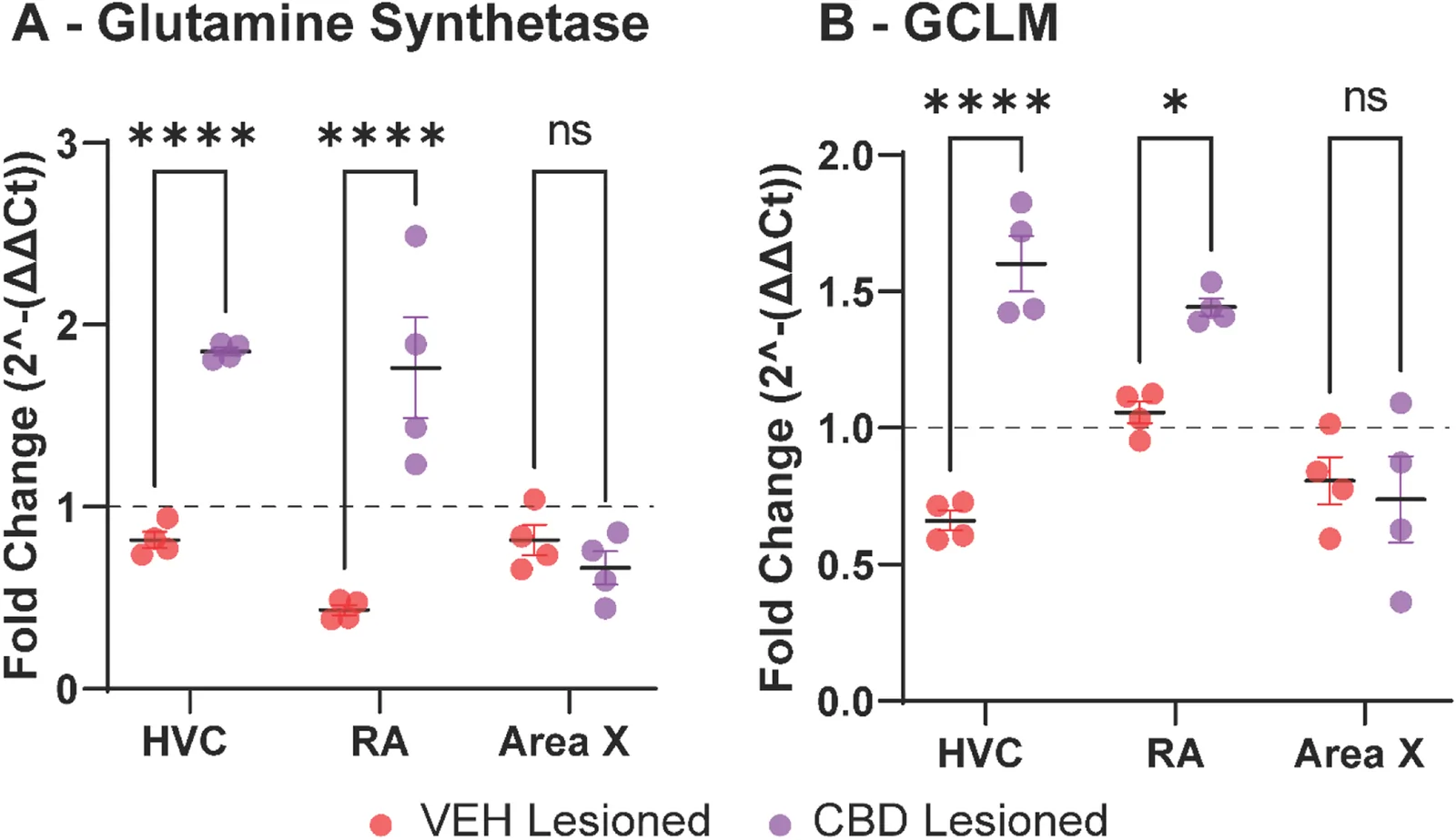
Cannabidiol enhances expression of astrocytic metabolic and antioxidant marker transcripts in song control nuclei following injury. Quantitative RT-PCR analysis of selected astrocyte-associated transcripts in the lesioned hemispheres of HVC, RA, and Area X from VEH- and CBD-treated birds, normalized to the contralateral unlesioned hemisphere. **(A)** Glutamine synthetase (GS), a key astrocytic enzyme supporting glutamate recycling and metabolic homeostasis, exhibited significantly increased mRNA expression in HVC and RA following CBD treatment. **(B)** Glutamate-cysteine ligase modifier subunit (GCLM), a regulator of glutathione synthesis and cellular antioxidant capacity, exhibited significantly increased mRNA expression in HVC and RA following CBD treatment. Data are presented as mean fold change ±SEM with individual data points representing biological replicates (n = 4 per group). Statistical comparisons were performed using mixed-effects ANOVA followed by Sidak’s multiple comparisons test (*p* < 0.05).

Messenger RNA expression was quantified from the same bulk-region punches using qRT-PCR and expressed as fold change relative to each animal’s contralateral control hemisphere. A two-way mixed-effects model revealed significant main effects of region (p = 0.0007) and treatment (p < 0.0001), along with a Region × Treatment interaction for glutamine synthetase (GS) mRNA (two-way model: Supplementary Table S2; *post hoc* comparisons; Supplementary Table S3). The Region × Treatment interaction indicates that CBD effects on astrocyte-associated metabolic gene expression are region-dependent. Consistent with this, *post hoc* comparisons revealed that CBD increased GS mRNA fold expression in HVC (VEH: 0.82-fold vs. CBD: 1.85-fold, 95% CI [−1.51-fold, −0.57-fold], p < 0.0001) and RA (VEH: 0.43-fold vs. CBD: 1.76-fold, 95% CI [−1.80-fold, −0.86-fold], p < 0.0001), with no effect in Area X (p > 0.05). Similarly, a model for GCLM mRNA revealed significant effects of region (p = 0.0001), treatment (p < 0.0001), and their interaction (p < 0.0001; Figure 9B). The Region × Treatment interaction indicates that CBD effects on astrocyte-associated antioxidant gene expression are also region-dependent. Post hoc comparisons revealed that CBD increased GCLM mRNA fold expression in HVC (VEH: 0.66-fold vs. CBD: 1.60-fold, 95% CI [−1.27-fold, −0.62-fold], p < 0.0001) and RA (VEH: 1.06-fold vs. CBD: 1.44-fold, 95% CI [−0.71-fold, −0.06-fold], p = 0.018), with no effect in Area X (p > 0.05). These findings indicate that CBD enhances astrocyte-mediated glutamate handling and antioxidant capacity in a region-dependent manner following injury.

### 3.8 CBD dampens astrocyte reactivity-associated transcriptional responses

Taken together, the combined changes in astrocyte percentages, diminished astrocyte apoptosis, normalization of lysosomal structure, and restoration of metabolic gene expression point toward coordinated modulation of astrocyte activation state. We therefore assessed reactivity-associated transcripts using bulk-region qRT-PCR, expressed as fold change relative to each animal’s contralateral control hemisphere (Figure 10). S100A10 mRNA was quantified as a marker of pan-reactive/generally anti-inflammatory astrocytes (Liddelow et al., 2017; Escartin et al., 2021), C3 mRNA as a marker most associated with neurotoxic astrocyte reactivity (Liddelow et al., 2017), and aromatase mRNA as an injury-responsive astrocyte marker linked to local estrogen synthesis and gliosis in the songbird brain (Duncan and Saldanha, 2011; Mehos et al., 2016; Pedersen et al., 2017). These transcripts were selected because they capture distinct aspects of the reactive astrocyte spectrum and have been shown to be modulated in injury models, allowing assessment of whether CBD shifts astrocyte phenotypes.

**FIGURE 10:**
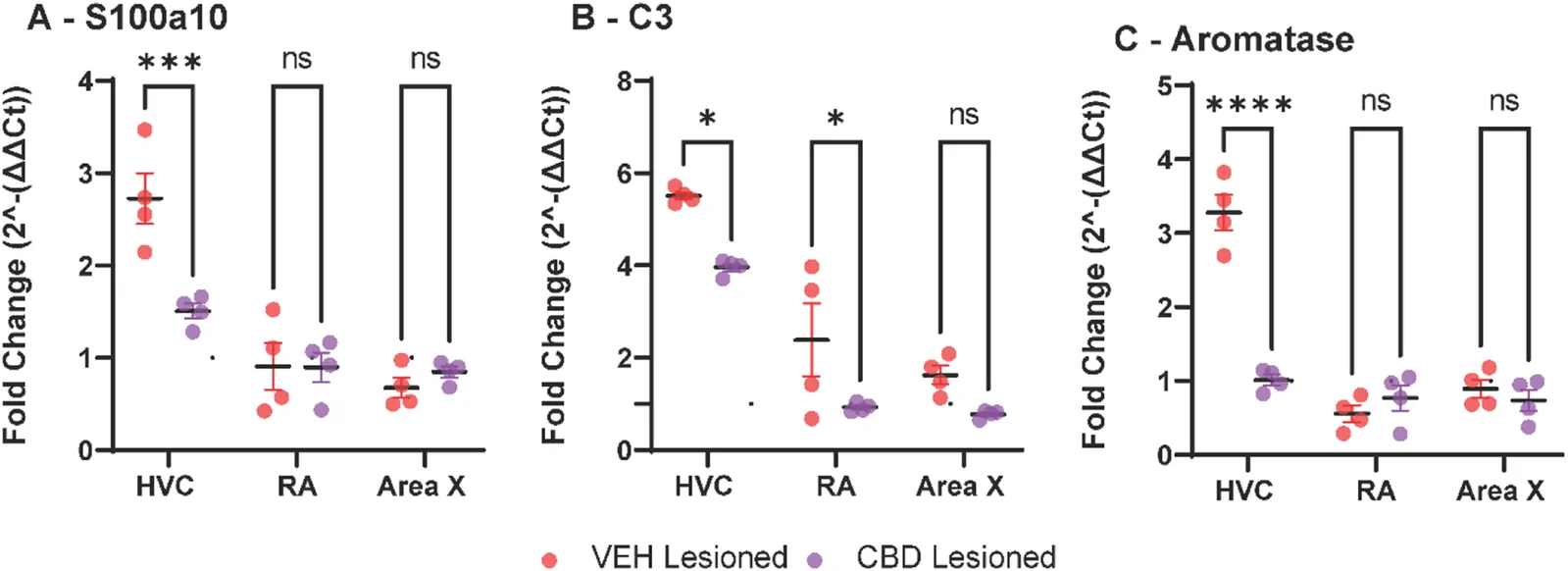
Cannabidiol attenuates injury-induced astrocyte reactivity markers in song control nuclei. Quantitative RT-qPCR analysis of selected astrocyte-associated transcripts in the lesioned hemispheres of HVC, RA, and Area X from VEH- and CBD-treated zebra finches, expressed as fold change relative to the contralateral unlesioned hemisphere and normalized to the reference gene TBP. **(A)** S100A10 (marker of anti-inflammatory/pan-reactive astrocytes) expression was significantly reduced in HVC following CBD treatment, with no significant changes observed in RA or Area X. **(B)** C3 (marker associated with neurotoxic A1-like astrocyte reactivity) expression was significantly reduced in both HVC and RA following CBD treatment. **(C)** Aromatase (injury-responsive astrocyte marker linked to local estrogen synthesis) expression was significantly reduced in HVC following CBD treatment. Data are presented as mean fold change ±SEM with individual data points representing biological replicates (n = 4 per group). Statistical comparisons were performed using mixed-effects ANOVA followed by Sidak’s multiple comparisons test (*p* < 0.05).

A two-way mixed-effects model revealed a Region × Treatment interaction for S100A10 mRNA (p = 0.0032; Figure 10A), indicating region-specific effects of CBD on astrocyte reactivity. Consistent with this, *post hoc* comparisons revealed that CBD reduced S100A10 mRNA fold expression in HVC (VEH: 2.73-fold vs. CBD: 1.51-fold, 95% CI [0.56-fold, 1.88-fold], p = 0.0004), with no effect in RA or Area X (p > 0.05). For C3 mRNA fold expression, significant main effects of region (p < 0.0001) and treatment (p = 0.0086) were observed (Figure 10B). Post hoc comparisons revealed that CBD reduced C3 mRNA expression in HVC (VEH: 5.51-fold vs. CBD: 3.96-fold, 95% CI [0.29-fold, 2.81-fold], p = 0.0136) and RA (VEH: 2.39-fold vs. CBD: 0.93-fold, 95% CI [0.20-fold, 2.72-fold], p = 0.0207), with no effect in Area X (p > 0.05). Aromatase mRNA (a marker of astrocyte reactivity) showed significant effects of region (p < 0.0001), treatment (p = 0.0025), and their interaction (p < 0.0001; Figure 10C), indicating region-dependent effects of CBD. Post hoc comparisons revealed that CBD reduced aromatase mRNA fold expression in HVC (VEH: 3.28-fold vs. CBD: 1.01-fold, 95% CI [1.70-fold, 2.84-fold], p < 0.0001), with no effect in RA or Area X (p > 0.05). Collectively, these findings indicate that CBD dampens astrocyte reactivity-associated gene expression, with the strongest effects in the lesioned HVC and its projection target RA; the regions showing the greatest injury-associated activation.

## 4 Discussion

This study follows from our earlier finding that cannabidiol (CBD; 10 mg/kg daily) protects vocal quality and accelerates recovery of learned song patterns following HVC microlesions in adult male zebra finches (Alalawi et al., 2019). The 10 mg/kg dose used here was selected based on that earlier full dose-response study, which also included sham-operated controls. Those experiments demonstrated that sham surgery produced negligible effects relative to the robust changes induced by focal HVC lesions. Vocal recovery in the CBD-treated groups was associated with profound suppression of neuroinflammation (reduced IL-6, IL-1β, and TNF-α), oxidative stress (reduced SOD2 expression and DHE fluorescence), microglial activation, and circuit-wide synaptic loss (preserved VGLUT2/PSD95 colocalization), as well as NRF2 pathway activation and upregulation of plasticity-related transcripts (BDNF, ARC/ARG3.1, MSK1) (Tripson et al., 2023).

The present experiments were designed to identify cellular substrates contributing to these protective effects. We now show that astrocyte-associated responses are a candidate cellular substrate of CBD-associated neuroprotection in the zebra finch song control circuit. These findings extend prior rodent TBI studies demonstrating general reductions in astrogliosis [e.g., 40] by providing region-specific evidence of preserved astrocyte viability, normalized lysosomal stress, suppressed reactivity programs (C3, S100A10, aromatase), and enhanced metabolic/antioxidant functions (GS, GCLM) across interconnected song nuclei. The present study therefore has a dual but staged focus: it examines acute glial correlates of CBD’s anti-neuroinflammatory actions, while placing those actions in the context of a circuit that supports recovery of learned vocal behavior. Speech and language recovery after TBI is the clinically relevant form of functional recovery this model is designed to inform. Together, the findings address a critical gap in post-TBI therapy: the need for well-tolerated, brain-penetrant agents capable of modulating neuroinflammation within behaviorally relevant CNS circuits, where traditional anti-inflammatories are limited by long-term toxicity and many biologics exhibit poor brain penetration.

A central finding is that HVC microlesions trigger substantial astrocyte apoptosis, which accounts for a large fraction of cleaved caspase-3-positive cells across the three song nuclei examined (Figures 3–6). CBD markedly reduced this astrocyte loss and preserved (or expanded) astrocyte populations in a region-dependent manner (Figure 6). This association between CBD treatment and astrocyte preservation is particularly relevant to secondary injury cascades, as the primary electrolytic lesion remains constant across treatment groups while CBD limits subsequent apoptotic, lysosomal, and reactivity responses that amplify circuit-wide dysfunction (Alalawi et al., 2019; Tripson et al., 2023). This pattern aligns with evidence that astrocyte viability is a critical determinant of circuit recovery after focal CNS injury (Gürer et al., 2009; Liu and Chopp, 2016; Alhadidi et al., 2024). The comparatively modest lesion effects on astrocyte percentage, neuronal percentage, and mean lysosomal area, despite robust increases in apoptosis, are expected at this 24-h time point. Quantification was restricted to non-infarcted fields, so surviving-cell percentages in spared neuropil can remain relatively stable even as a subset of cells enter apoptosis. Lysosomal area in vehicle-treated birds was also similar across hemispheres, indicating that enlargement was not the dominant 24-h phenotype and that CBD-associated reductions were largely treatment-driven rather than simple reversals of a large lesion-only increase. The strongest CBD effects were observed in HVC, the site of the primary electrolytic injury, with smaller or more selective changes in its efferent targets RA and Area X, consistent with local secondary injury plus limited transsynaptic propagation at 24 h.

Astrocytes support neuronal survival and synaptic stability through metabolic coupling (lactate shuttling, glutamate homeostasis), redox buffering, and structural support (Gürer et al., 2009; Alhadidi et al., 2024). By preserving these core functions, CBD likely mitigates secondary neuronal vulnerability and supports the synaptic remodeling and/or preservation required for vocal recovery. The observed increases in GS and GCLM expression further suggest enhanced glutamate recycling and glutathione-mediated antioxidant capacity, consistent with the synaptic preservation documented in our prior work (Tripson et al., 2023).

Injury also induced significant lysosomal stress in astrocytes (enlarged LAMP1^+^ lysosomes and upregulated LAMP1 and LC3 transcripts; Figures 7, 8), which CBD effectively normalized. While these changes are consistent with improved autophagic clearance of damaged components (Bylicky et al., 2018), they could also reflect reduced generation of cellular debris upstream of autophagy due to CBD’s broader anti-inflammatory and anti-oxidative actions (Tripson et al., 2023). In RA, CBD reduced LAMP1 and LC3 mRNA to values below the contralateral comparator (fold change <1). Because these data are normalized to the unlesioned hemisphere rather than to an uninjured naïve control, this pattern indicates suppression relative to the within-animal comparator, not a demonstrated fall below true baseline. We interpret the RA decrease as consistent with attenuation of injury-associated lysosomal/autophagy transcription, but we do not claim that values below 1 are themselves beneficial; autophagic flux assays (e.g., LC3-II turnover in the presence of lysosomal inhibitors) will be required to distinguish reduced demand from impaired clearance.

CBD additionally suppressed injury-induced astrocyte reactivity markers (C3, S100A10, aromatase; Figure 10). Coordinated downregulation of these transcripts suggests that CBD prevents diversion of astrocytic resources into stress programs, thereby sustaining core homeostatic functions. This profile is consistent with CBD-mediated inhibition of NF-κB-driven transcription via PPARγ and TRPV1 signaling (Atalay Ekiner et al., 2022; Puighermanal et al., 2024) and aligns with the broad anti-inflammatory actions observed previously (Tripson et al., 2023).

Region-specific preservation of neurons was most evident in directly-lesioned HVC (Figures 3, 6) raising the possibility that CBD may support not only neuronal survival but also adult neurogenesis or neuronal recruitment. This effect may be mediated, at least in part, by astrocyte-derived trophic support. Future experiments incorporating proliferation and neurogenesis markers (e.g., BrdU, DCX) will be required to directly test this possibility (Wang et al., 2002; Esposito et al., 2011; Brenowitz and Larson, 2015; Scarante et al., 2020).

A limitation of our current study is that a single 24-h post-lesion time point was used. This interval was chosen to capture acute secondary injury processes and early glial responses, but it was insufficient for most birds to recover singing behavior. Consequently, the current experiments could not evaluate the relationship between the observed astrocytic changes and longer-term functional recovery of learned song. Future work employing multiple recovery time points will be required to define the temporal dynamics of CBD’s effects on astrocyte viability, lysosomal stress, reactivity programs, and circuit function. Although n = 4 birds per group is a modest sample for the immunofluorescence and qRT-PCR endpoints, the within-subject contralateral control design, repeated-measures structure, and large observed effect sizes (partial ηp^2^ = 0.37–0.98; Supplementary Table S2) provided high statistical power (>0.90) for the effects reported here. A second limitation is that qRT-PCR was performed on whole-nucleus punches; transcriptional changes reported for LAMP1, LC3, GS, GCLM, S100A10, C3, and aromatase are therefore region-level inferences rather than direct astrocyte-specific measurements. Finally, while the zebra finch HVC microlesion model provides high translational relevance for sensorimotor circuit injury after TBI, possible species differences in glial responses and cannabinoid pharmacology should be considered when translating findings to mammalian TBI.

Collectively, these results identify astrocyte-associated responses as a candidate cellular component of CBD-associated neuroprotection in the injured song circuit. The present 24-h experiment does not establish astrocytes as a causal mechanism of protection, nor does it demonstrate accelerated behavioral recovery. Speech and language are learned sensorimotor skills supported by specialized circuits that are vulnerable to TBI; partial HVC injury in the zebra finch is used because it produces a measurable deficit in an analogous circuit after a standardized focal lesion (Li et al., 2000; Jarvis, 2004; Prather et al., 2017). These findings therefore bear on whether early glial responses after focal injury are a tractable target for protecting that class of circuit (Aychman et al., 2023; Dong et al., 2024).

## Data Availability

The datasets presented in this study can be found in online repositories. The names of the repository/repositories and accession number(s) can be found in the article/Supplementary Material.
