# Supplementary material for: Cannabidiol preserves astrocyte viability and attenuates injury-induced glial stress responses across zebra finch song control nuclei
Source: Front Pharmacol. 2026 Oct 9;17:1972787. doi: 10.3389/fphar.2026.1972787 (PMC13690509; doi:10.3389/fphar.2026.1972787)
Supplement: Supplementary file 1 [file DataSheet1.pdf]

SUPPLEMENTARY MATERIAL

1. Supplementary Methods

Syllable Segmentation and Cluster Movement Analysis Methods.

Continuous song recordings were imported into Sound Analysis Pro 2011 (SAP, [30]) and segmented into individual syllables using automated amplitude- and entropy-based thresholding, with constraints on minimum syllable duration and inter-syllable gap. Thresholds were optimized per bird and held constant between pre- and post-lesion recordings. The present study employed unilateral HVC microlesions. The 24 h post-lesion time point was selected because our previous *bilateral* HVC lesion study demonstrated the greatest divergence in song disruption between CBD- and VEH-treated birds, as measured by Kullback–Leibler (K–L) distance, while song in both treatment groups remained significantly different from pre-lesion baseline at that time point. Because unilateral lesions produce more modest and potentially differently timed behavioral deficits than bilateral lesions, the applicability of this timepoint to the present unilateral design is an assumption rather than a directly established result.

Of 30 birds that sang during the recording period, 13 produced at least 800 syllables during the 24 h post-lesion recording period and met the inclusion criterion for analysis (VEH, n = 7; CBD, n = 6). SAP-derived acoustic features were exported for cluster analysis.

Syllable data were imported into a custom Python application. Fourteen SAP-derived acoustic features — syllable duration, mean amplitude, mean pitch, mean frequency modulation (FM), mean amplitude modulation (AM), mean entropy, mean pitch goodness, mean frequency, and the variances of pitch, frequency modulation, entropy, pitch goodness, frequency, and amplitude modulation — were z-score normalized across the pooled pre- and post-lesion dataset for each bird. Syllables were then clustered simultaneously using a distance-based, threshold-defined nearest-neighbor connectivity algorithm: pairwise Euclidean distances in the pooled z-scored feature space were computed via a KD-tree radius query using a fixed threshold of 1.3, syllable pairs within this threshold distance were connected in a graph, and clusters were defined as the resulting connected components — a method equivalent to single-linkage hierarchical clustering [31]. Pre- and post-lesion labels were withheld during cluster formation. Clusters containing fewer than 100 pooled syllables were treated as unclustered residuals and excluded from centroid and displacement calculations. Two-dimensional principal components analysis (PCA) projections were used only for visualization of the pooled clusters and were not used to perform the clustering itself.

For each retained cluster present at both time points, pre- and post-lesion centroids were calculated as the mean feature vector (in the same z-scored feature space used for clustering) of that cluster's syllables at each time point. Cluster displacement was defined as the Euclidean distance between the pre- and post-lesion centroids. Each cluster was assigned a weight equal to its pooled (pre + post) syllable count divided by the total number of syllables across all retained clusters. Syllable Cluster Movement (SCM) was then calculated as the weighted mean displacement across all clusters present at both time points, with weights renormalized to sum to 1 across only those clusters (i.e., clusters missing a time point were excluded from both the numerator and denominator rather than contributing zero). An SCM of 0 indicates no change in syllable acoustic structure, with larger values reflecting greater post-lesion displacement of syllable clusters. Because SCM is defined only over clusters present at both time points, it does not capture syllables that reorganized into entirely new post-lesion clusters or disappeared post-lesion; this is addressed further as a limitation below.

2. Supplementary Results

CBD decreased post-lesion syllable cluster displacement.

Syllable Cluster Movements (SCM) for the 13 birds meeting the inclusion criteria out of 30 birds recorded (VEH, $n = 7$; CBD, $n = 6$) are presented in Fig. S2. An unpaired t-test showed CBD-treated birds had lower SCM than VEH-treated birds (VEH: 0.821 vs. CBD: 0.582; 95% CI [−0.479, −0.0001]; $p = 0.0499$), a difference that was statistically significant but only marginally so, given that the confidence interval bound approached zero. This result suggests possible preservation of pre-lesion syllable cluster organization following HVC microlesion, though given the small sample size and borderline p-value, it should be interpreted cautiously pending replication in a larger cohort.

Behavioral analysis was limited to a single 24 h post-lesion time point, representing a study limitation because additional post-lesion time points would provide a more comprehensive assessment of lesion-induced changes and recovery. Furthermore, because unilateral HVC microlesions produce more modest behavioral deficits than bilateral lesions, treatment-related differences are correspondingly smaller, and only 13 of the 30 birds recorded met the inclusion criterion, further limiting statistical power. The SCM metric itself is also limited to clusters detectable at both time points, potentially underestimating disruption in birds with more substantial post-lesion reorganization of syllable structure. Although CBD reduced SCM in the present study, future studies examining larger cohorts would improve the ability to resolve behavioral differences following unilateral lesions and more precisely estimate the magnitude of CBD-mediated protection.

3. Supplementary Figures

Figure S1: Representative pre-to-post-lesion syllable cluster movement analysis
Figure S2: Cannabidiol reduces pre-to-post-lesion syllable cluster movement
Figure S3: Images of lysosomal LAMP1 localization in glutamine synthetase-positive astrocytes
Figure S4: Apoptotic (cleaved caspase-3-positive) glutamine synthetase-positive astrocytes
Figure S5: Representative images of secondary-only control sections
Figure S6: Melt curve analysis confirms amplification specificity of qPCR targets

A. (Brown 9) - VEH

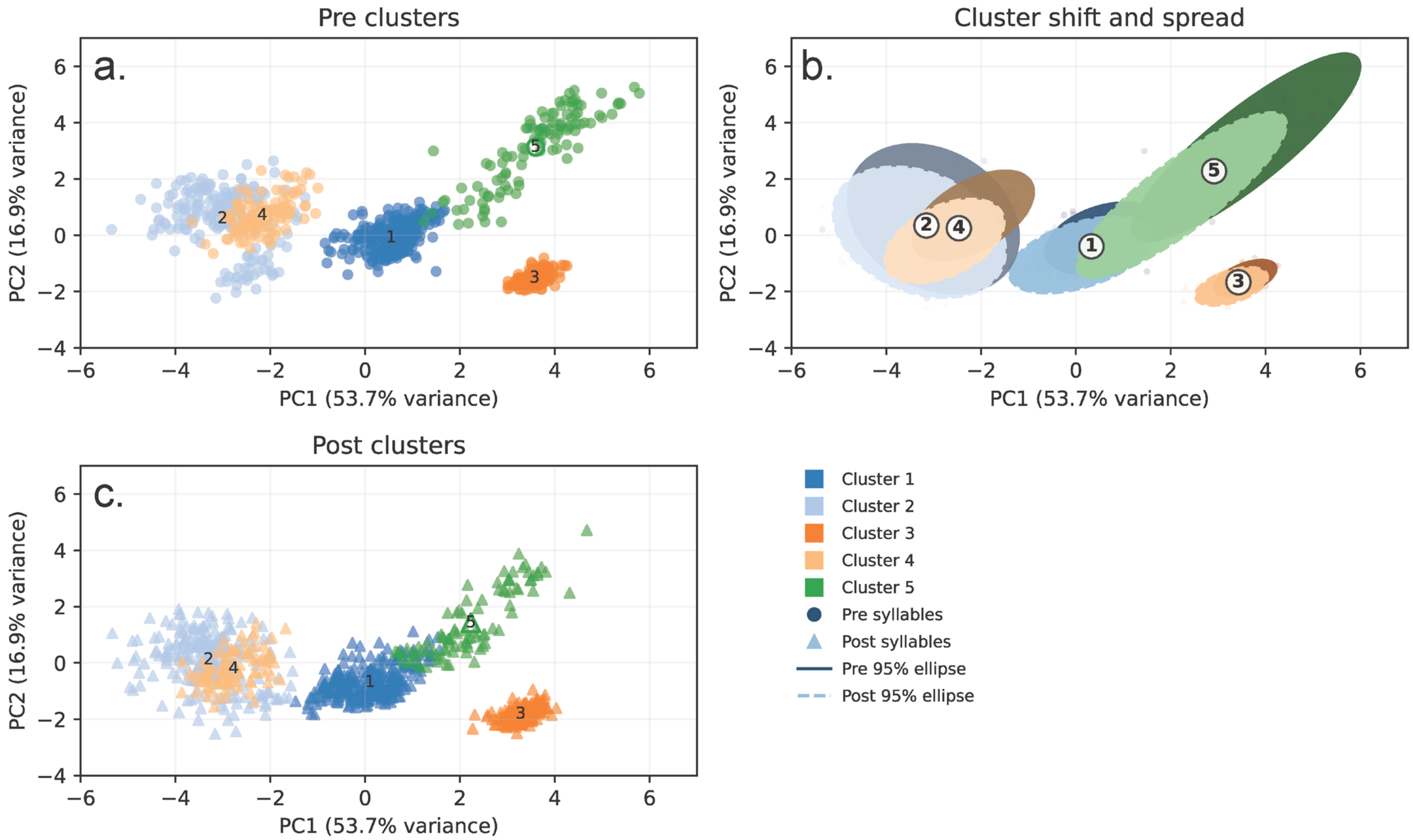

B. (Blue 26) - CBD

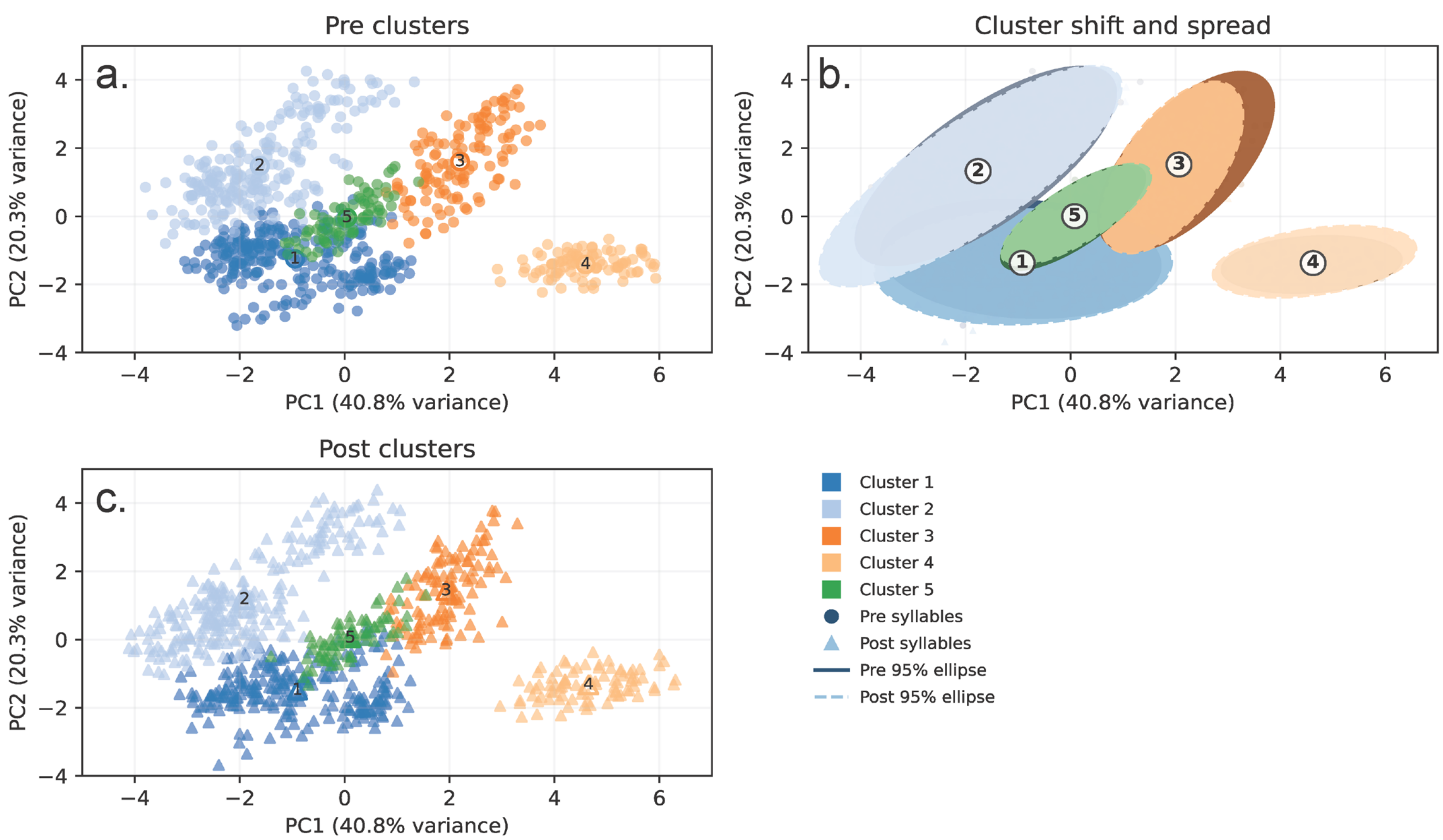

Figure S1. Representative pre-to-post-lesion syllable cluster movement analysis. Representative examples of pre-to-post-lesion syllable cluster movement in a vehicle-treated (VEH) animal (A; Brown 9) and a cannabidiol-treated (CBD) animal (B; Blue 26). Within each treatment group, (a) shows the distribution of pre-lesion syllables following unsupervised clustering, (b) shows the overlaid pre- and post-lesion cluster distributions to illustrate cluster movement and dispersion, and (c) shows the distribution of post-lesion syllables. Clustering was performed on pooled pre- and post-lesion syllables for each animal, allowing acoustically similar syllables from both recordings to be assigned to a common set of clusters. For each individual bird, principal component analysis (PCA) was performed independently on the pooled pre- and post-lesion syllable dataset to visualize the multidimensional acoustic feature space; consequently, the principal components and the percentage of variance explained differ between birds. Colored symbols represent individual syllables assigned to each cluster, with cluster identities indicated by color. Circular symbols denote pre-lesion syllables, triangular symbols denote post-lesion syllables, solid ellipses represent the 95% covariance ellipses of pre-lesion clusters, and dashed ellipses represent the corresponding post-lesion clusters. Greater separation and reduced overlap between corresponding pre- and post-lesion cluster distributions indicate greater pre-to-post-lesion syllable cluster movement

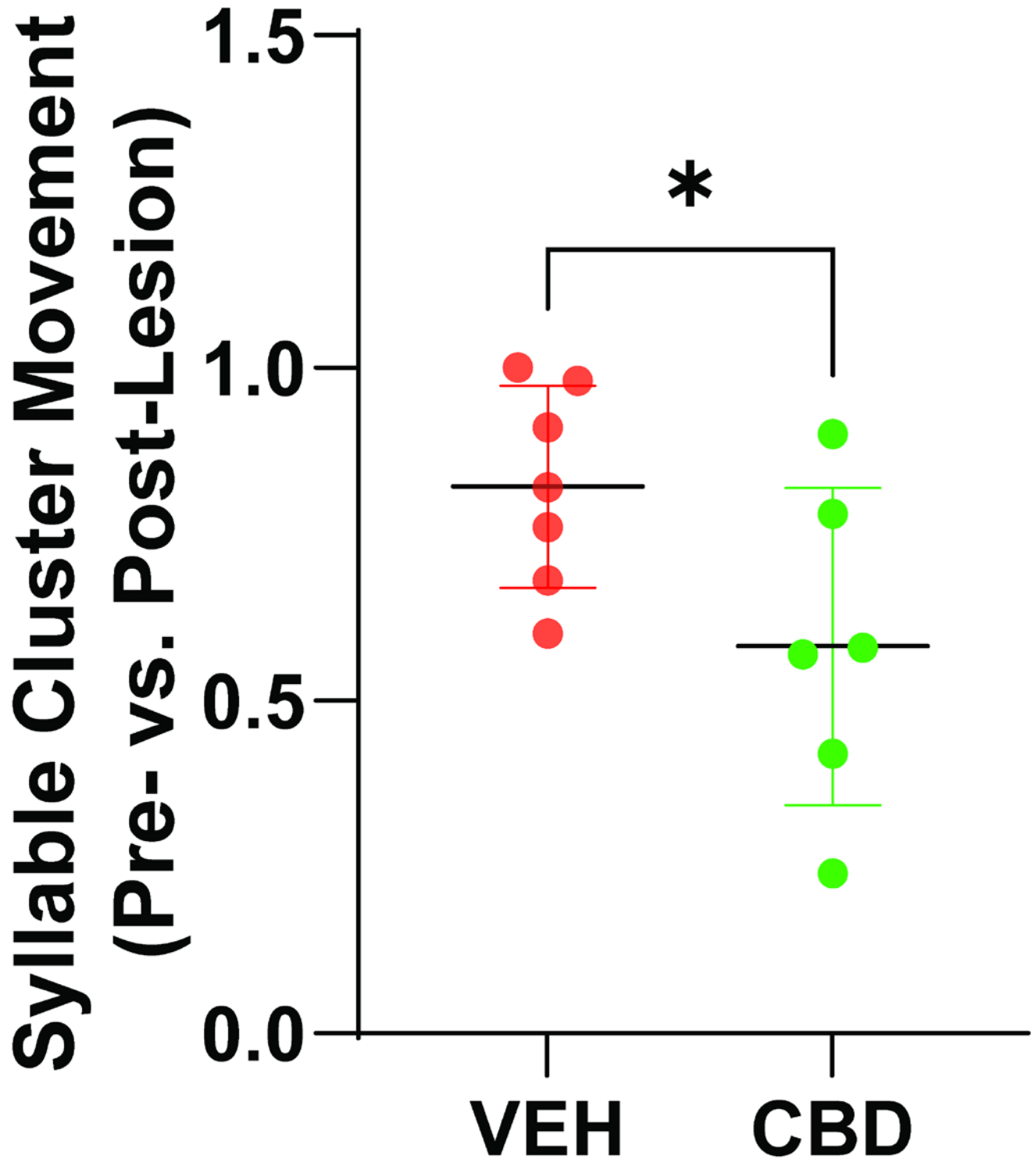

Figure S2. Cannabidiol reduces pre-to-post-lesion syllable cluster movement. Group-level quantification of pre-to-post-lesion syllable cluster movement following unilateral HVC lesion. Pre-to-post-lesion syllable cluster movement was calculated by measuring the Euclidean displacement between corresponding syllable cluster centroids from pre-lesion and post-lesion recordings and weighting each cluster according to its relative abundance within the pooled syllable dataset. A SCM value of 0 indicates no detectable shift in syllable cluster centroids, whereas progressively larger values indicate greater changes in acoustic syllable organization following lesion. Representative examples of the clustering workflow and centroid displacement analysis are shown in Fig. S1. Data are presented as mean ± SD with individual data points representing biological replicates (VEH, n = 7; CBD, n = 6). Red symbols indicate VEH-treated animals and green symbols indicate CBD-treated animals. CBD-treated animals exhibited lower SCM values than VEH-treated animals (p = 0.0499). Statistical comparisons were performed using an unpaired two-tailed t-test ($p < 0.05$).

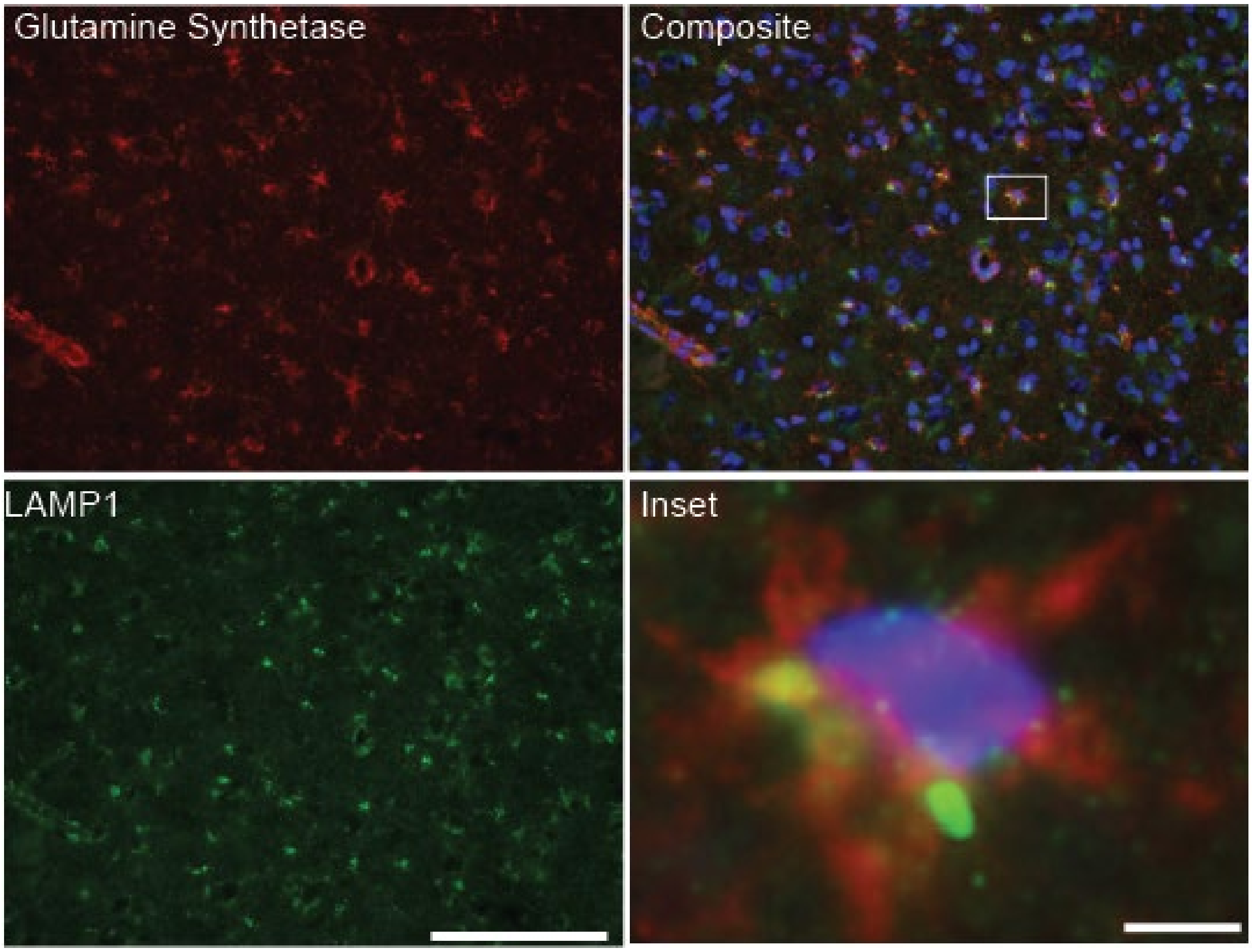

Figure S3. Images of lysosomal LAMP1 localization in glutamine synthetase-positive astrocytes. Representative images of glutamine synthetase (GS; red) LAMP1 (green) and Hoechst (blue) labeling in zebra finch brain tissue. GS immunoreactivity highlights astrocytic cell bodies and proximal processes (top left 40X) while LAMP1 labeling identifies lysosomal structures (bottom left 40X). The merged image (top right 40X) illustrates the spatial relationship between astrocytes and lysosomal compartments. The boxed region denotes the area shown at higher magnification (bottom right 263× digital zoom). The inset demonstrates the subcellular distribution of LAMP1 signal within GS-positive astrocytes with larger LAMP1-positive structures predominantly localized within GS-positive cells. Scale bars: 100 µm (top left top right bottom left) 10 µm (bottom right).

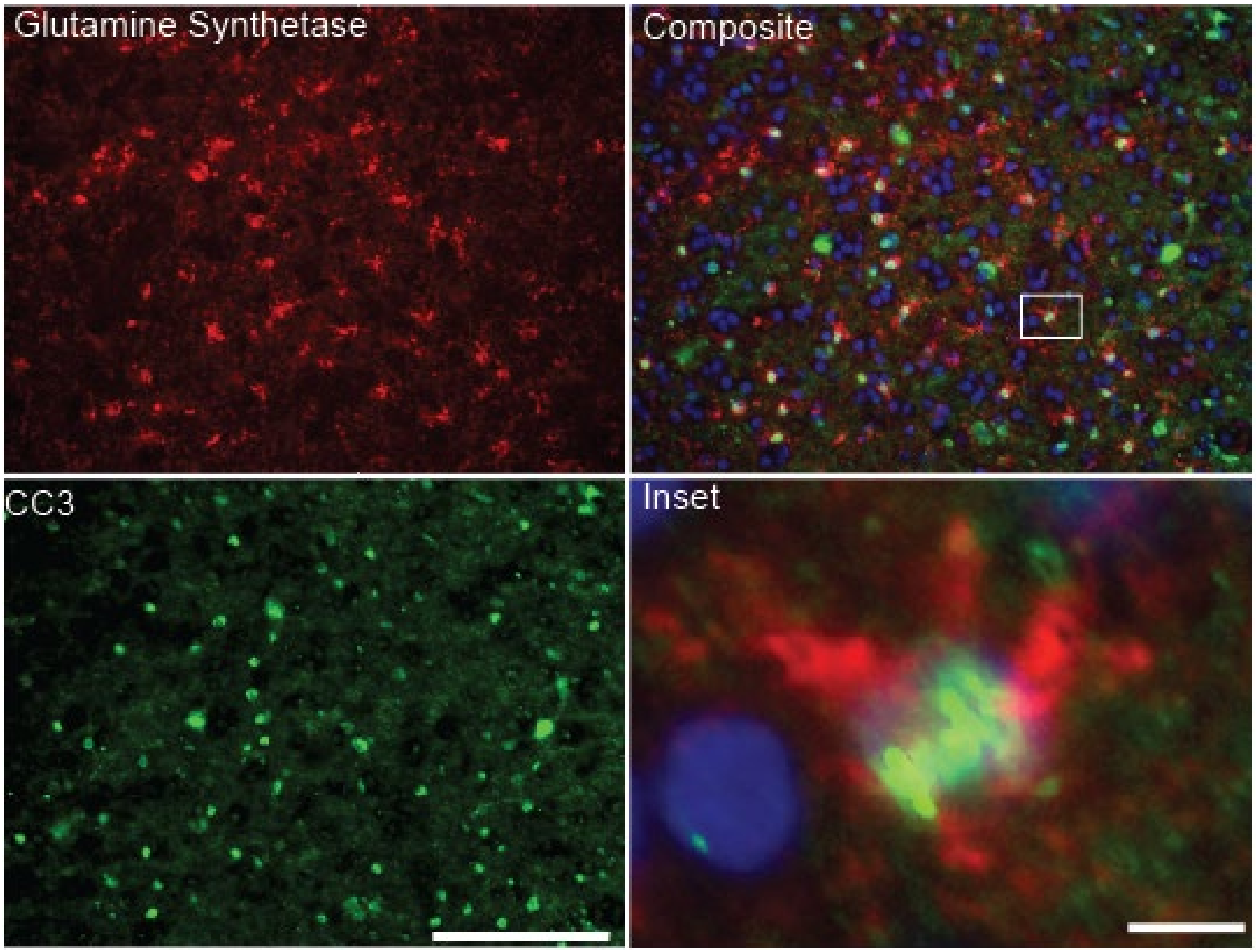

Figure S4. Apoptotic (cleaved caspase-3-positive) glutamine synthetase-positive astrocytes. Representative images of glutamine synthetase (GS; red) cleaved caspase-3 (CC3; green) and Hoechst (blue) labeling in zebra finch brain tissue. GS immunoreactivity highlights astrocytic cell bodies and proximal processes (top left 40X) while CC3 labeling identifies apoptotic cells (bottom left 40X). The merged image (top right 40X) illustrates the spatial relationship between CC3 expression and astrocytes. The boxed region denotes the area shown at higher magnification (bottom right 263× digital zoom). Most CC3-positive cells are localized within GS-positive astrocytes. Scale bars: 100 µm (top left top right bottom left) 10 µm (bottom right).

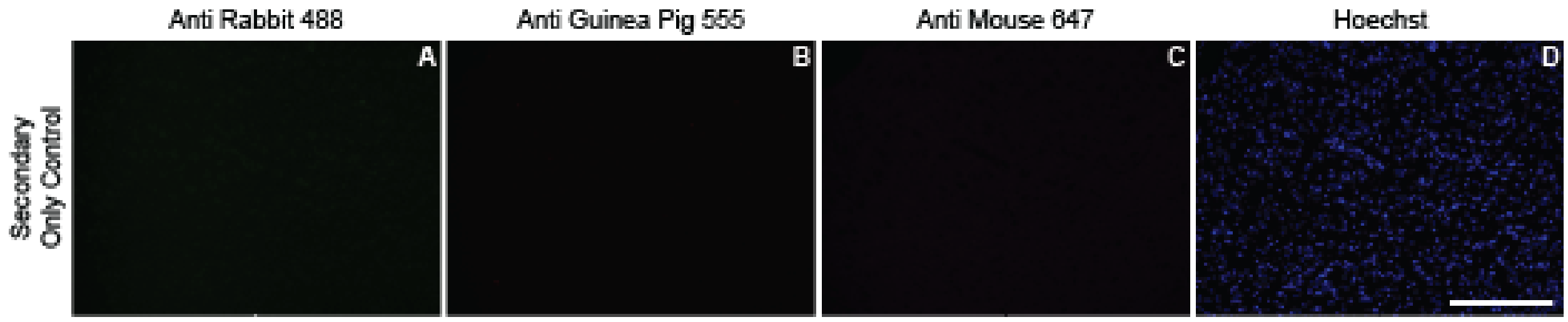

Figure S5. Representative images of secondary-only control sections. Representative images of secondary-only control sections (20X scale bar = 200 μm). Sections were incubated with secondary antibodies only including goat anti-rabbit Alexa Fluor 488 (Thermo Fisher Scientific Cat. #A-11008 1:500) (A) goat anti-guinea pig Alexa Fluor 555 (Thermo Fisher Scientific Cat. #A-21435 1:500) (B) and goat anti-mouse Alexa Fluor 647 (Thermo Fisher Scientific Cat. #A-21235 1:500) (C) with nuclei counterstained using Hoechst (D). Lipofuscin autofluorescence was quenched using TrueBlack Lipofuscin Autofluorescence Quencher (Cell Signaling Technology Cat. #92401 1:20) which reduces lipofuscin-associated and general background fluorescence. Minimal background fluorescence was observed across all channels confirming specificity of primary antibody labeling.

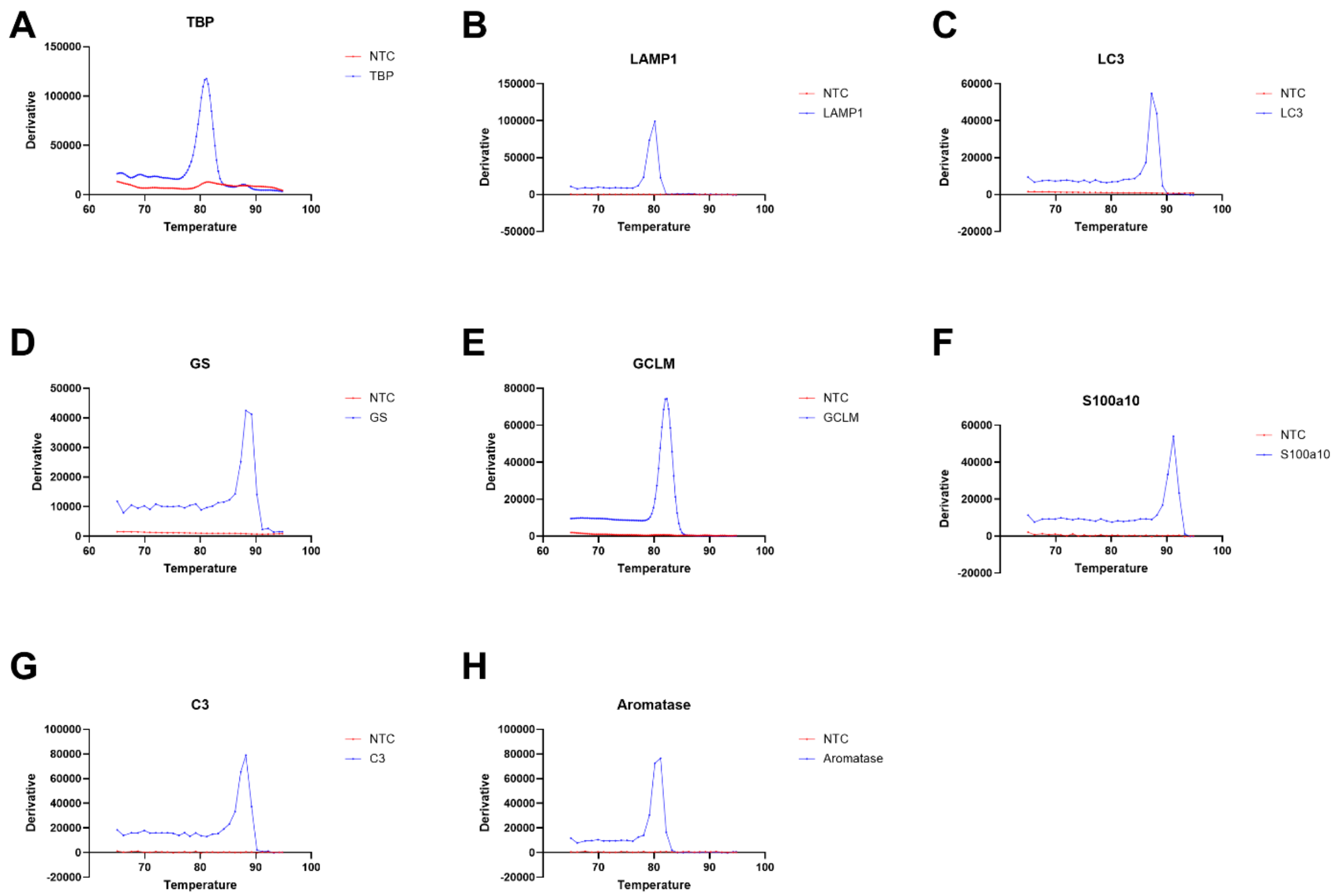

Figure S6. Melt curve analysis confirms amplification specificity of qPCR targets. Panels correspond to: (A) TATA binding protein (TBP), (B) LAMP1, (C) LC3, (D) GS, (E) GCLM, (F) S100a10, (G) C3, and (H) Aromatase. For each gene, a single, sharp melt peak was observed in reactions containing cDNA template, indicating amplification of a single specific product. In contrast, no distinct melt peak was observed in no-template controls (NTC), confirming the absence of non-specific amplification or primer-dimer formation

4. Supplementary Tables

Table S1: Primers used for quantitative RT-PCR
Table S2: Summary of main effects and interactions from mixed-effects models ANOVAs
Table S3: Region-specific post hoc comparisons across treatment and lesion conditions

Table S1. Primers used for quantitative RT-PCR

Gene Symbol	Accession Number	Forward Primer	Reverse Primer
TBP	XM_030268321.4	TCACACACCAGCAGTTCAGC	CTGCTCGTACTTTAGCACCAGTTA
GS	XM_030279644.4	AATCTGCTCCCCAGCTTCCC	TCAGCCTTTCCAGAGCAGATCA
GCLM	XM_030279191.4	GACTGAGTGGAGCTCAAAGGT	CCAGGTCAACTGCGTCTCTG
LAMP1	XM_002191571.5	TGCATCAACTCCAAGCACATC	AGAGACCCTATCTTCAGCACATTC
LC3B	NM_001245293.2	AGCTTCCAGTTTTGGATAAGACC	TTCTCGCTCTCGTACACCTC
CYP19A1	AH008871.2	CTCCACCGACAAAAATCCAC	GGGTTTCCAGGACCATCTTT
S100a10	XM_030291396.4	TCATGGACAAGGAGTTCCCG	TGCTGCACGAAGTAGTCGTT
C3	XM_072920066.1	GGCTCTCCCAGCAGAACTAC	AGAACCGACACGTCCAACTC

Table S2. Summary of main effects and interactions from mixed-effects models ANOVAs.

Figure	Effect	F (DFn, DFd)	p-value	Partial η_p^2
6A	Region	F(2, 12) = 30.69	<0.0001	0.84
6A	Lesion condition	F(1, 6) = 0.4152	0.5432	0.07
6A	Treatment	F(1, 6) = 48.33	0.0004	0.89
6A	Region × Lesion condition	F(2, 12) = 9.122	0.0039	0.60
6A	Region × Treatment	F(2, 12) = 8.061	0.006	0.57
6A	Lesion condition × Treatment	F(1, 6) = 1.994	0.2076	0.25
6A	Region × Lesion condition × Treatment	F(2, 12) = 2.347	0.1379	0.28
6B	Region	F(2, 12) = 252.8	<0.0001	0.98
6B	Lesion condition	F(1, 6) = 15.65	0.0075	0.72
6B	Treatment	F(1, 6) = 2.078	0.1995	0.26
6B	Region × Lesion condition	F(2, 12) = 6.610	0.0116	0.52
6B	Region × Treatment	F(2, 12) = 10.61	0.0022	0.64
6B	Lesion condition × Treatment	F(1, 6) = 0.1862	0.6811	0.03
6B	Region × Lesion condition × Treatment	F(2, 12) = 3.044	0.0853	0.34
6C	Region	F(2, 12) = 15.22	0.0005	0.72
6C	Lesion condition	F(1, 6) = 120.7	<0.0001	0.95
6C	Treatment	F(1, 6) = 39.35	0.0008	0.87
6C	Region × Lesion condition	F(2, 12) = 14.03	0.0007	0.70
6C	Region × Treatment	F(2, 12) = 0.1776	0.8395	0.03
6C	Lesion condition × Treatment	F(1, 6) = 24.17	0.0027	0.80
6C	Region × Lesion condition × Treatment	F(2, 12) = 1.176	0.3417	0.16
6D	Region	F(2, 12) = 5.755	0.0177	0.49
6D	Lesion condition	F(1, 6) = 8.513	0.0267	0.59
6D	Treatment	F(1, 6) = 1.853	0.2223	0.24
6D	Region × Lesion condition	F(2, 12) = 9.148	0.0039	0.60
6D	Region × Treatment	F(2, 12) = 4.351	0.0379	0.42
6D	Lesion condition × Treatment	F(1, 6) = 3.154	0.1261	0.35
6D	Region × Lesion condition × Treatment	F(2, 12) = 5.408	0.0212	0.47
7D	Region	F(2, 12) = 5.333	0.022	0.47
7D	Lesion condition	F(1, 6) = 11.36	0.015	0.65
7D	Treatment	F(1, 6) = 42.47	0.0006	0.88
7D	Region × Lesion condition	F(2, 12) = 0.3589	0.7057	0.06
7D	Region × Treatment	F(2, 12) = 0.8693	0.4441	0.13
7D	Lesion condition × Treatment	F(1, 6) = 8.026	0.0298	0.57
7D	Region × Lesion condition × Treatment	F(2, 12) = 5.793	0.0173	0.49

8A	Region	F(2, 18) = 162.9	<0.0001	0.95
8A	Treatment	F(1, 18) = 30.12	<0.0001	0.63
8A	Region × Treatment	F(2, 18) = 19.13	<0.0001	0.68
8B	Region	F(2, 18) = 117.9	<0.0001	0.93
8B	Treatment	F(1, 18) = 10.40	0.0047	0.37
8B	Region × Treatment	F(2, 18) = 27.50	<0.0001	0.75
9A	Region	F(2, 18) = 11.23	0.0007	0.56
9A	Treatment	F(1, 18) = 51.09	<0.0001	0.74
9A	Region × Treatment	F(2, 18) = 19.29	<0.0001	0.68
9B	Region	F(2, 18) = 15.92	0.0001	0.64
9B	Treatment	F(1, 18) = 34.23	<0.0001	0.66
9B	Region × Treatment	F(2, 18) = 16.51	<0.0001	0.65
10A	Region	F(2, 12) = 37.39	<0.0001	0.86
10A	Treatment	F(1, 6) = 5.312	0.0607	0.47
10A	Region × Treatment	F(2, 12) = 9.643	0.0032	0.62
10B	Region	F(2, 12) = 84.37	<0.0001	0.93
10B	Treatment	F(1, 6) = 14.71	0.0086	0.71
10B	Region × Treatment	F(2, 12) = 0.8347	0.4577	0.12
10C	Region	F(2, 12) = 70.95	<0.0001	0.92
10C	Treatment	F(1, 6) = 24.82	0.0025	0.81
10C	Region × Treatment	F(2, 12) = 47.72	<0.0001	0.89

Partial η_p^2 was calculated as $\eta_p^2 = (F \times df\_numerator) / (F \times df\_numerator + df\_denominator)$. Values of ≈0.01, 0.06, and 0.14 represent small, medium, and large effects, respectively.

Table S3. Region-specific post hoc comparisons across treatment and lesion conditions

Figure	Region	Comparison	Mean Difference	95% CI	Adjusted p-value
6A	HVC	VEH Un-lesioned vs. VEH Lesioned	-1.217	-3.735 to 1.301	0.4237
6A	HVC	CBD Un-lesioned vs. CBD Lesioned	-4.131	-6.649 to -1.612	0.0025
6A	HVC	VEH Un-lesioned vs. CBD Un-lesioned	-2.414	-4.932 to 0.1047	0.0606
6A	HVC	VEH Lesioned vs. CBD Lesioned	-5.327	-7.845 to -2.809	0.0003
6A	RA	VEH Un-lesioned vs. VEH Lesioned	-0.3385	-5.167 to 4.490	0.9752
6A	RA	CBD Un-lesioned vs. CBD Lesioned	1.487	-3.341 to 6.315	0.6366
6A	RA	VEH Un-lesioned vs. CBD Un-lesioned	-9.619	-14.60 to -4.635	0.0007
6A	RA	VEH Lesioned vs. CBD Lesioned	-7.793	-12.78 to -2.809	0.0036
6A	Area X	VEH Un-lesioned vs. VEH Lesioned	5.016	1.275 to 8.757	0.0147
6A	Area X	CBD Un-lesioned vs. CBD Lesioned	1.351	-2.390 to 5.092	0.5462
6A	Area X	VEH Un-lesioned vs. CBD Un-lesioned	-0.6725	-4.503 to 3.158	0.8858
6A	Area X	VEH Lesioned vs. CBD Lesioned	-4.338	-8.169 to -0.5068	0.0269
6B	HVC	VEH Un-lesioned vs. VEH Lesioned	-12.69	-26.24 to 0.8679	0.0638
6B	HVC	CBD Un-lesioned vs. CBD Lesioned	-13.45	-27.01 to 0.1033	0.0515
6B	HVC	VEH Un-lesioned vs. CBD Un-lesioned	-14.02	-27.98 to -0.0695	0.0489
6B	HVC	VEH Lesioned vs. CBD Lesioned	-14.79	-28.74 to -0.8342	0.0378
6B	RA	VEH Un-lesioned vs. VEH Lesioned	6.07	-4.046 to 16.19	0.2364
6B	RA	CBD Un-lesioned vs. CBD Lesioned	-5.677	-15.79 to 4.439	0.2739
6B	RA	VEH Un-lesioned vs. CBD Un-lesioned	4.293	-6.305 to 14.89	0.5396
6B	RA	VEH Lesioned vs. CBD Lesioned	-7.455	-18.05 to 3.143	0.1859

6B	Area X	VEH Un-lesioned vs. VEH Lesioned	-14.36	-28.13 to -0.6018	0.0624
6B	Area X	CBD Un-lesioned vs. CBD Lesioned	-6.989	-20.75 to 6.773	0.3335
6B	Area X	VEH Un-lesioned vs. CBD Un-lesioned	-0.8486	-13.03 to 11.34	0.9809
6B	Area X	VEH Lesioned vs. CBD Lesioned	6.526	-5.658 to 18.71	0.3544
6C	HVC	VEH Un-lesioned vs. VEH Lesioned	-32.81	-43.26 to -22.37	0.0002
6C	HVC	CBD Un-lesioned vs. CBD Lesioned	-9.774	-20.22 to 0.6739	0.0640
6C	HVC	VEH Un-lesioned vs. CBD Un-lesioned	6.407	-4.772 to 17.58	0.3096
6C	HVC	VEH Lesioned vs. CBD Lesioned	29.45	18.27 to 40.63	<0.0001
6C	RA	VEH Un-lesioned vs. VEH Lesioned	-21.25	-42.57 to 0.0655	0.0498
6C	RA	CBD Un-lesioned vs. CBD Lesioned	-6.914	-28.23 to 14.41	0.6085
6C	RA	VEH Un-lesioned vs. CBD Un-lesioned	7.94	-10.54 to 26.42	0.5018
6C	RA	VEH Lesioned vs. CBD Lesioned	22.28	3.802 to 40.76	0.019
6C	Area X	VEH Un-lesioned vs. VEH Lesioned	-53.11	-63.75 to -42.47	<0.0001
6C	Area X	CBD Un-lesioned vs. CBD Lesioned	-24.23	-34.87 to -13.59	0.0010
6C	Area X	VEH Un-lesioned vs. CBD Un-lesioned	0.515	-10.05 to 11.08	0.9906
6C	Area X	VEH Lesioned vs. CBD Lesioned	29.4	18.83 to 39.96	<0.0001
6D	HVC	VEH Un-lesioned vs. VEH Lesioned	-14.51	-25.04 to -3.984	0.0130
6D	HVC	CBD Un-lesioned vs. CBD Lesioned	-2.587	-13.12 to 7.942	0.7446
6D	HVC	VEH Un-lesioned vs. CBD Un-lesioned	0.1046	-9.089 to 9.298	0.9995
6D	HVC	VEH Lesioned vs. CBD Lesioned	12.03	2.836 to 21.22	0.0117
6D	RA	VEH Un-lesioned vs. VEH Lesioned	-1.634	-8.937 to 5.670	0.7816
6D	RA	CBD Un-lesioned vs. CBD Lesioned	-1.063	-8.366 to 6.241	0.8988
6D	RA	VEH Un-lesioned vs. CBD Un-lesioned	-0.1096	-6.634 to 6.415	0.9989
6D	RA	VEH Lesioned vs. CBD Lesioned	0.4611	-6.063 to 6.985	0.9803

6D	Area X	VEH Un-lesioned vs. VEH Lesioned	-2.082	-4.921 to 0.7570	0.1408
6D	Area X	CBD Un-lesioned vs. CBD Lesioned	-0.7842	-3.623 to 2.055	0.6919
6D	Area X	VEH Un-lesioned vs. CBD Un-lesioned	-1.063	-3.541 to 1.415	0.5030
6D	Area X	VEH Lesioned vs. CBD Lesioned	0.2352	-2.243 to 2.713	0.9649
7D	HVC	VEH Un-lesioned vs. VEH Lesioned	-0.3163	-2.096 to 1.463	0.8540
7D	HVC	CBD Un-lesioned vs. CBD Lesioned	-1.815	-3.595 to -0.0359	0.0463
7D	HVC	VEH Un-lesioned vs. CBD Un-lesioned	4.86	2.407 to 7.313	0.0006
7D	HVC	VEH Lesioned vs. CBD Lesioned	3.361	0.9073 to 5.814	0.0088
7D	RA	VEH Un-lesioned vs. VEH Lesioned	1.347	-1.050 to 3.745	0.2729
7D	RA	CBD Un-lesioned vs. CBD Lesioned	-2.677	-5.074 to -0.2797	0.0324
7D	RA	VEH Un-lesioned vs. CBD Un-lesioned	4.973	2.649 to 7.297	0.0003
7D	RA	VEH Lesioned vs. CBD Lesioned	0.9488	-1.375 to 3.273	0.5347
7D	Area X	VEH Un-lesioned vs. VEH Lesioned	-1.501	-3.383 to 0.3807	0.1094
7D	Area X	CBD Un-lesioned vs. CBD Lesioned	-0.9359	-2.818 to 0.9458	0.3464
7D	Area X	VEH Un-lesioned vs. CBD Un-lesioned	3.051	1.073 to 5.028	0.0039
7D	Area X	VEH Lesioned vs. CBD Lesioned	3.616	1.638 to 5.594	0.0011
8A	HVC	VEH Lesioned vs. CBD Lesioned	0.7235	0.4153 to 1.0320	<0.0001
8A	RA	VEH Lesioned vs. CBD Lesioned	0.6066	0.2984 to 0.9148	0.0002
8A	Area X	VEH Lesioned vs. CBD Lesioned	-0.2166	-0.5248 to 0.0916	0.2238
8B	HVC	VEH Lesioned vs. CBD Lesioned	0.6258	0.3482 to 0.9034	<0.0001
8B	RA	VEH Lesioned vs. CBD Lesioned	0.3913	0.1137 to 0.6689	0.0048
8B	Area X	VEH Lesioned vs. CBD Lesioned	-0.4278	-0.7054 to -0.1503	0.0022
9A	HVC	VEH Lesioned vs. CBD Lesioned	-1.035	-1.505 to -0.5655	<0.0001

9A	RA	VEH Lesioned vs. CBD Lesioned	-1.329	-1.798 to -0.8586	<0.0001
9A	Area X	VEH Lesioned vs. CBD Lesioned	0.1529	-0.3170 to 0.6228	0.7874
9B	HVC	VEH Lesioned vs. CBD Lesioned	-0.9411	-1.268 to -0.6139	<0.0001
9B	RA	VEH Lesioned vs. CBD Lesioned	-0.3868	-0.7140 to -0.0596	0.0180
9B	Area X	VEH Lesioned vs. CBD Lesioned	0.0677	-0.2595 to 0.3949	0.9324
10A	HVC	VEH Lesioned vs. CBD Lesioned	1.219	0.5593 to 1.8790	0.0004
10A	RA	VEH Lesioned vs. CBD Lesioned	0.0095	-0.6505 to 0.6694	>0.9999
10A	Area X	VEH Lesioned vs. CBD Lesioned	-0.1713	-0.8312 to 0.4886	0.8774
10B	HVC	VEH Lesioned vs. CBD Lesioned	1.549	0.2904 to 2.8070	0.0136
10B	RA	VEH Lesioned vs. CBD Lesioned	1.457	0.1988 to 2.7160	0.0207
10B	Area X	VEH Lesioned vs. CBD Lesioned	0.8456	-0.4129 to 2.1040	0.2564
10C	HVC	VEH Lesioned vs. CBD Lesioned	2.268	1.698 to 2.838	<0.0001
10C	RA	VEH Lesioned vs. CBD Lesioned	-0.2136	-0.7835 to 0.3563	0.7087
10C	Area X	VEH Lesioned vs. CBD Lesioned	0.1575	-0.4124 to 0.7274	0.8565

Adjusted p-values were calculated using Tukey's multiple comparisons test for immunofluorescence analyses and Šídák's multiple comparisons test for qPCR analyses.
